# Radiolytic Elimination of Nabumetone from Aqueous Solution: Degradation Efficiency, and Degradants’ Toxicity

**DOI:** 10.3390/molecules30010064

**Published:** 2024-12-27

**Authors:** Ivana Tartaro Bujak, David Klarić, Bono Lučić, Krunoslav Bojanić, Maro Bujak, Nives Galić

**Affiliations:** 1 Radiation Chemistry and Dosimetry Laboratory, Ruđer Bošković Institute, Bijenička cesta 54, 10000 Zagreb, Croatia; itartaro@irb.hr; 2 Department of Chemistry, Faculty of Science, University of Zagreb, Horvatovac 102a, 10000 Zagreb, Croatia; dklaric@chem.pmf.hr; 3 NMR Centre, Ruđer Bošković Institute, Bijenička cesta 54, 10000 Zagreb, Croatia; lucic@irb.hr; 4 Laboratory for Aquaculture Biotechnology, Ruđer Bošković Institute, Bijenička cesta 54, 10000 Zagreb, Croatia; krunoslav.bojanic@irb.hr (K.B.); maro.bujak@irb.hr (M.B.)

**Keywords:** nabumetone, ionizing radiation, high-resolution mass spectrometry, degradation products, toxicity

## Abstract

Advanced oxidation processes (AOPs), including ionizing radiation treatment, are increasingly recognized as an effective method for the degradation of pharmaceutical pollutants, including non-steroidal anti-inflammatory drugs (NSAIDs). Nabumetone (NAB), a widely used NSAID prodrug, poses an environmental risk due to its persistence in aquatic ecosystems and its potential toxicity to non-target organisms. In this study, the radiolytic degradation of NAB was investigated under different experimental conditions (dose rate, radical scavenging, pH, matrix effect), and the toxicity of its degradation products was evaluated. NAB was rapidly degraded at 300 Gy with prolonged irradiation. Mineralization of about 88% of NAB solutions was observed based on the evaluation of total organic carbon (TOC). The most efficient degradation of NAB occurred under N_2_O conditions, while it was retarded in the presence of thiourea. The water matrix components had a significant influence on the efficiency of degradation. In addition, the main degradation products were identified by LC-HRMS. Toxicity studies on different bacteria showed no significant impact of the NAB degradation products, while in silico predictive methods revealed their slightly increased toxicity compared to the parent compound, but considerably lower toxicity in comparison to its main active form 6-methoxy-2-naphthylacetic acid (MNA). Additionally, significantly lower toxicities are predicted for degradation products in N_2_O saturated solution. These results underline the importance of optimizing irradiation parameters for effective degradation and minimizing the formation of harmful by-products. Understanding all aspects of the AOP processes and the toxicological effects of the degradation products ensures effective mitigation of potential environmental and health risks of water treatment processes.

## 1. Introduction

In recent years, the world has been confronted with increasing water pollution caused by numerous harmful substances. These include pharmaceuticals, engineered nanomaterials, food additives, hormones, steroids, personal care products, and veterinary medicines. Pharmaceuticals in particular play a crucial role in human health but their widespread use has led to their presence in the environment. It is estimated that around (30–90)% of pharmaceuticals currently in use end up in the environment in their original form or as metabolites [1]. In addition, up to 30% of unused pharmaceuticals are improperly disposed, further contributing to environmental pollution. Non-steroidal anti-inflammatory drugs (NSAIDs) are among the most commonly used pharmaceuticals in the world [2]. As a result, NSAIDs have been detected in various environmental compartments, mainly in water bodies, soils, and sediments [3]. Diclofenac is at the top of the environmental “watch lists” due to its widespread detection in aquatic environments and its significant ecological risks. It has been added to in the European Union’s Water Framework Directive watch list of substances requiring EU-wide monitoring, due to its effects on aquatic organisms, particularly fish. It has raised concerns about bioaccumulation and toxicity even at low concentrations. Other NSAIDs such as ibuprofen, naproxen, and ketoprofen have also been extensively studied and are frequently monitored due to their persistence and potential impact on aquatic ecosystems [4,5,6,7,8].

Nabumetone is one of the NSAID drugs that have been used for more than three decades for the treatment of osteoarthritis [9]. It has non-acidic and lipophilic properties. NAB is a pro-drug and its main active form used for the treatment of the disease is the metabolite 6-methoxy-2-naphthylacetic acid (MNA) [9]. MNA can no longer be metabolized by the human body and is therefore excreted into the water environment together with NAB. Like many pharmaceuticals, NAB can also enter the wastewater systems via various routes. These include human excretion after ingestion, improper disposal of unused medicines, and industrial discharge. As it is a less common NSAID, relatively little attention has been paid to its study. Recently, it has been reported that NAB and MNA are frequently detected in surface waters across China, with concentrations ranging from 17.3 to 290.1 ng/L and 14.2 to 355.6 ng/L for NAB and MNA, respectively [10]. The presence of these substances in water at high levels can pose ecological hazards to aquatic life and potential health risks to humans, especially with chronic exposure [11]. Kapuścińska and co-authors have reported a serious ecotoxicological risk studied in green algae from the presence of NAB and flufenamic acid [12]. Further research is needed to clarify these risks and to enable more effective regulation and management of pharmaceutical pollutants in the aquatic environment.

Conventional wastewater and drinking water treatment processes are not able to completely remove NAB and MNA. Therefore, the development of effective post-treatment technologies to eliminate these compounds or to transform them so that the products are less toxic is important and imperative [10]. To address this issue, advanced oxidation processes (AOPs) have emerged as promising technologies for the degradation of pharmaceuticals like NAB. AOPs utilize reactive oxygen species, such as hydroxyl radicals, to break down complex organic molecules into smaller, less harmful substances. In general, techniques such as photocatalysis, ozonation, and Fenton reactions are effective in degrading NSAIDs under controlled conditions, but further research is needed to optimize the processes, increase their efficiency and reduce their release into the environment [13,14,15]. Ionizing radiation treatment is an innovative method within AOPs that offers a promising, unique and environmentally friendly approach to the effective removal of drugs through their degradation. It stands out from other AOPs because no additional chemicals are added; it can be used effectively at room temperature, and it is insensitive to color and suspended solids and can break down recalcitrant compounds through reactive species formed in situ during radiolysis of the water [16,17]. To the best of our knowledge, there is only one publication reporting the degradation of NAB during the UV/monochloroamine process [10]. Therefore, very little attention has been paid to the removal of NAB by ionizing radiation as AOP, although radiation technology is now applicable on an industrial scale [18], which underlines the importance of this study.

In the present study, the degradation of NAB using radiation technology was systematically investigated as an alternative, environmentally friendly method. This study is a contribution to the existing literature on irradiation of aqueous NAB solutions under various experimental conditions. In addition, our study summarizes all possible reactions occurring during radiolysis of an aqueous solution of NAB, the degradation efficiency of NAB, the biological toxicity, and toxicity predictions of the degradation products after treatment identified by LC-HR MS/MS technique.

## 2. Results and Discussion

### 2.1. Concentration Effect and Dose Rate Influence

The degradation of water pollutants, such as pharmaceuticals, by gamma irradiation, can occur by direct and/or indirect means. It usually begins with the formation of primary reactive species from the radiolysis of water. These include hydrogen atoms (^●^H), hydrated electrons e^−^_aq_, hydroxyl radicals (^●^OH), and the less reactive species H_3_O^+^, as shown in Equation (1).

H_2_O⟿(0.28) e^−^_aq_, (0.062) ^●^H, (0.28) ^●^OH, (0.047) H_2_, (0.073) H_2_O_2_, (0.28) H_3_O^+^ (1)

The values in the brackets represent the G-values of each species in units of μmol/J. Since radiolysis produces strongly oxidizing and reducing species, the degradation of pharmaceuticals can take place by both oxidative and reductive means. The degradation of a pharmaceutical compound can be defined as the concentration change per unit dose, in a selected dose interval. The NAB concentration was determined by HPLC-DAD method. The decrease of its concentration is exponential and follows the pseudo first-order kinetic model according to Equation (2):

−ln(*c*/*c*_0_) = *kD* (2)

**Figure 1:**
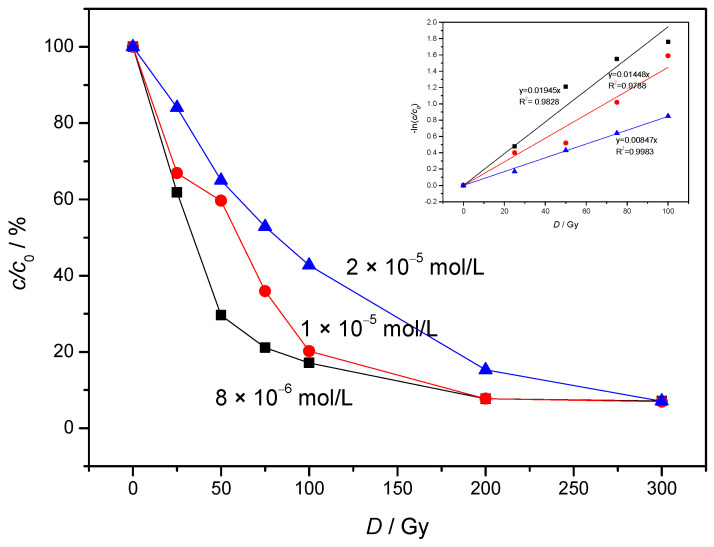
Removal of NAB at different initial concentrations versus absorbed dose under air equilibrium conditions. (*P* = 0.4 Gy/s, pH = 6.39, different symbols used for different NAB concentration).

NAB was examined in three different concentrations by ionizing radiation. NAB was degraded by about 93% at all three concentrations after applying 300 Gy. It was found that the concentration of NAB decreases with the absorbed dose (Figure 1). The degradation rate of NAB was faster at lower concentrations of NAB. The rate constant *k* decreased from 0.01945 to 0.0084 Gy^−1^ with an increase in the initial concentration of NAB from 8 × 10^−6^ to 2 × 10^−5^ mol/L. The above results indicate that there is a strong dependence of the initial concentration on the dose constant. These results also signify that, at lower *c*_0_ of NAB, a lower dose was required to achieve 50% and 90% of the decomposition level of NAB with correspondingly higher values of the dose constant and vice versa.

The obtained *k* value was utilized to determine the doses needed to achieve 90% (*D*_0.90_) degradation of NAB using Equation (3):

*D*_0.90_ = ln10/*k* (3)

At initial concentrations of 2 × 10^−5^, 1 × 10^−5^ and 8 × 10^−6^ mol/L the required doses for 90% of NAB removal were 271, 159 and 118 Gy respectively.

**Figure 2:**
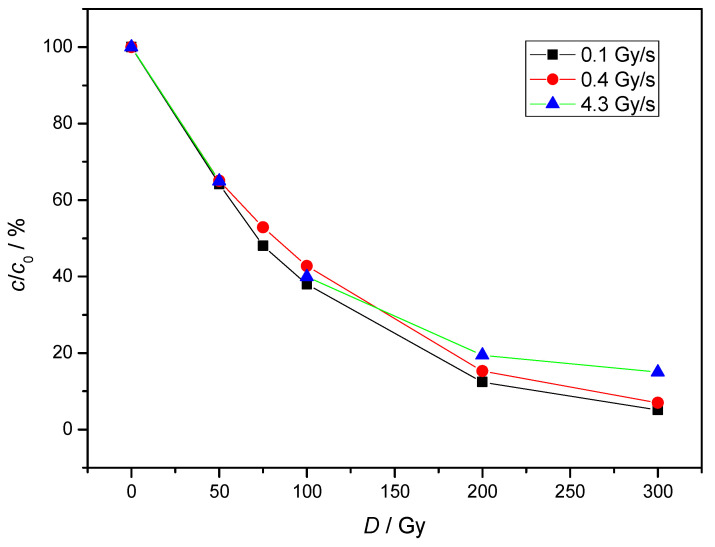
The influence of dose rate on the degradation of NAB under air equilibrium conditions. (*c*(NAB) = 2 × 10^−5^ mol/L; pH = 6.39, different symbols used for different dose rates).

Regarding the effect of the dose rate, several studies [19,20,21] have shown that higher dose rates generally improve the efficiency of pharmaceutical degradation. This is attributed to the increased concentration of reactive radicals generated at a steady state, which leads to an increased reaction rate. The decomposition curve of NAB by γ-radiation at different dose rates is shown in Figure 2. In our system, the difference between the dose rates was greatest at higher absorbed doses. At an absorbed dose of 300 Gy, the degradation of NAB increased from 85% to 93% when the dose rate decreased from 4.3 Gy/s to 0.4 Gy/s. Under experimental conditions, self-recombination reactions of radicals occurred which reduced the efficiency of NAB degradation at a higher dose rate (Table 1, Equations (4)–(6)). In the continuation of our work, the NAB concentration was 2 × 10^−5^ mol/L, and the experiments were performed at 0.4 Gy/s.

### 2.2. The Influence of Different Radicals and Scavengers

**Figure 3:**
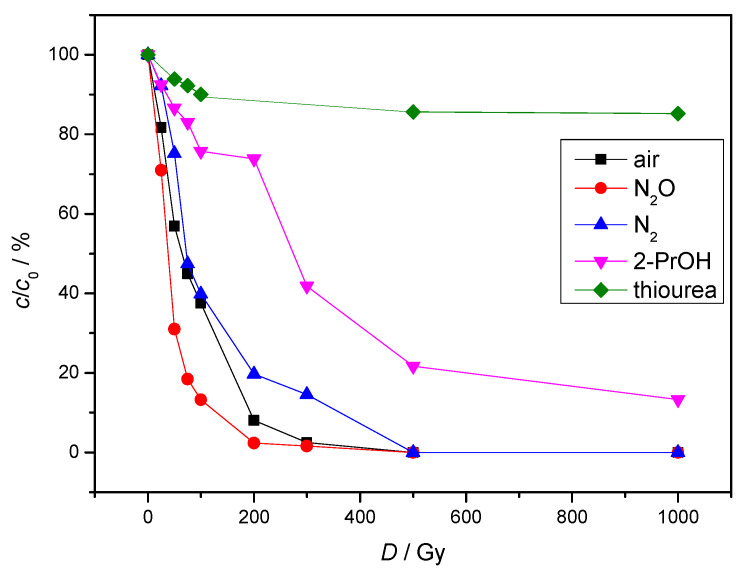
Radiolytic degradation of aqueous solution of NAB saturated with N_2_O, N_2_, in equilibrium with air and in the presence of 2-PrOH and thiourea. (*c*(NAB) = 2 × 10^−5^ mol/L; *c*(2-PrOH) = 0.1 mol/L; *c*(thiourea) = 3 × 10^−3^ mol/L; *P* = 0.4 Gy/s; pH = 6.39).

**Figure 4:**
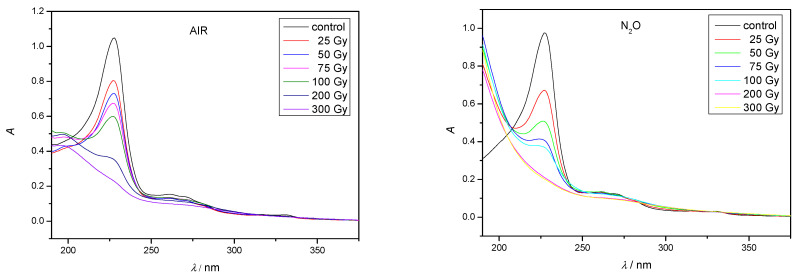
UV-Vis spectra of NAB in air and N_2_O saturated aqueous solutions after irradiation. (*c*(NAB) = 2 × 10^−5^ mol/L; *P* = 0.4 Gy/s).

By changing experimental conditions such as adding scavengers to the aqueous solution we gained information which radicals were the most responsible for degradation of NAB. During the radiolysis of aqueous solutions saturated with nitrogen (N_2_), both hydroxyl radicals (^●^OH) and hydrated electrons (e^−^_aq_) are present. The gamma-radiolytic degradation of nitrogen-saturated aqueous solutions of NAB proceeds slower than under equilibrium conditions with air (Figure 3). The results can be attributed to mutual interactions of the main products of water radiolysis ^●^OH and e^−^_aq_ (Table 1, Equation (6)) which leads to a decrease in the interactions with NAB. The NAB degradation can be easily seen from the UV-Vis spectra. As an example, the UV-Vis spectra of NAB in air and N_2_O saturated solutions are given in Figure 4, and those with N_2_ or 2-propanol in Figures S1 and S2.

To obtain oxidation conditions, the entire amount of e^−^_aq_ must be converted to ^●^OH, which is achieved by purging the aqueous solution with nitrous oxide (N_2_O). Figure 4 shows that the gamma-radiolytic degradation of NAB aqueous solutions saturated with N_2_O is faster compared to equilibrium conditions with air. The results obtained can be attributed to the fact that the concentration of hydroxyl radicals almost doubles (Table 1, Equation (8)). Moreover, in aromatic compounds, the reaction with hydroxyl radicals starts with ring hydroxylation and the further reaction with reactive oxygen species leads to the formation of open conjugated structures. Therefore, hydroxyl radicals have a significant influence on the NAB degradation as its structure contains naphthalene moiety. To investigate the influence of e^−^_aq_, the removal of ^●^OH radicals is necessary. Various radical scavengers such as 2-PrOH and thiourea are used for this purpose. 2-PrOH is a very strong radical scavenger for hydrogen atoms (^●^H) and hydroxyl radicals (^●^OH) and produces the less reactive radical ^●^CH_2_CCH_3_OH (Table 1, Equations (9) and (10)). As expected, the gamma radiolytic degradation of NAB aqueous solutions with the addition of 2-PrOH is significantly slower compared to equilibrium conditions with air (Figure 4). Thus, hydrated electrons have a significantly lower influence on the NAB degradation in comparison to hydroxyl radicals.

Thiourea is a very fast scavenger of all three main radicals formed during the radiolysis of water, ^●^OH, ^●^H and e^−^_aq_. Therefore, the gamma-radiolytic degradation of NAB aqueous solutions with the addition of thiourea was the slowest.

### 2.3. Effect of the Initial Solution pH

**Figure 5:**
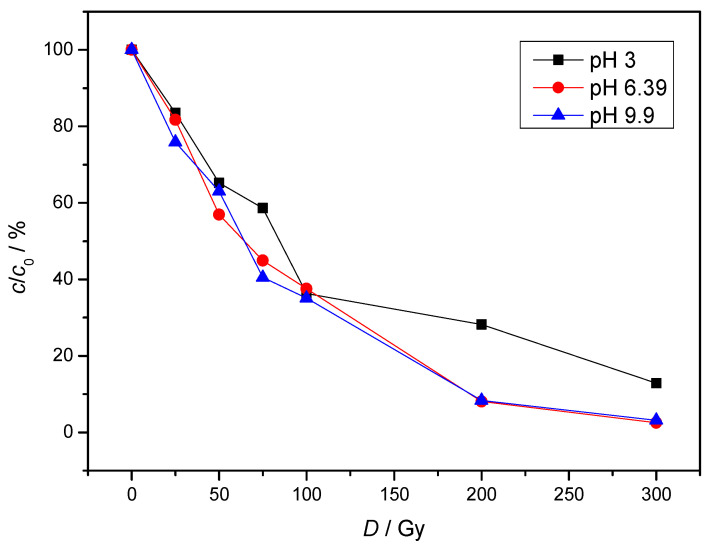
The influence of pH on the degradation of NAB under air equilibrium conditions. (*c*(NAB) = 2 × 10^−5^ mol/L, *P* = 0.4 Gy/s).

The pH value is a decisive parameter that influences the degradation behavior of pollutants. The efficiency of an oxidation system is highly dependent on the pH value, as it influences the behavior of the oxidants, *G*-value of the reactive species, the activity and the solubility of the reaction components. The results of the NAB degradation in various pH are shown in Figure 5. The pH varied from 3 to almost 10, with slower degradation under acidic conditions than under neutral and alkaline conditions. The removal rate of NAB was very high after irradiation with 300 Gy (87%, 98% and 97%).

The NAB solubility is not pH dependent since NAB is a nonionizable drug, so the slower degradation in acidic conditions can be attributed to media. Under acidic conditions, the formation of ^●^H occurs (Table 1, Equation (11)). By increasing the concentration of ^●^H radicals, they tend to undergo the recombination reaction shown in Equation (4). This reduces the ^●^OH concentration, which further slows down NAB degradation.

In the pH range 6 to 8 ^●^H radicals react with ^−^OH and the concentration of e^−^_aq_ (Equation (12)) is increasing, which explains higher NAB degradation. In general, degradation of pollutants is favored at weak acid to neutral conditions and decreases at strong acid conditions.

### 2.4. Inorganic Ions Influence and Matrix Effect

Inorganic anions are ubiquitous in the water body and many studies confirm [20,24,26] that the inorganic anions also have an important influence on the performance of AOPs. Inorganic anions react with reactive species which leads to the transformation of reactive species.

**Figure 6:**
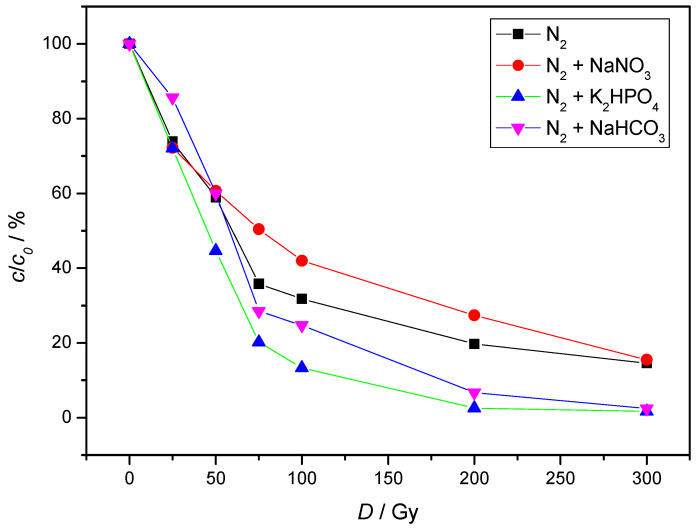
Influence of inorganic salt ions on the radiolytic degradation of NAB. (*c*(NAB) = 2 × 10^−5^ mol/L, *c*(NaHCO_3_) = 1.9 mmol/L, *c*(NaNO_3_) = 1 mmol/L, *γ*(K_2_HPO_4_) = 7 mg/L; *P* = 0.4 Gy/s).

The results obtained from chromatographic measurements (Figure 6) show that nitrates have the greatest influence on the gamma-radiolytic degradation of NAB. The results obtained can be attributed to the fact that nitrate is a scavenger of hydrated electrons (Table 1, Equation (13)) and reacts with H^+^ ions, leading to the formation of nitric acid (Table 1, Equation (14)). HNO_3_ also reacts with ^●^OH and ^●^H radicals (Table 1, Equations (15) and (16)). By reducing concentration of ^●^OH and ^●^H radicals, the number of collisions of the primary water radiolysis products with NAB molecules decreases.

Furthermore, the results showed that at higher doses the presence of HCO_3_^−^ and HPO_4_^2−^ promotes the degradation of NAB. HCO_3_^−^ is often considered as a radical scavenger due to its reaction with ^●^OH (Table 1, Equation (17)). In our system, its presence had an opposite effect, confirming a more complex role. Although formed carbonate radicals have lower redox potential than the hydroxyl radicals, they may have better removal performance in the degradation of organic pollutants [27,28,29] This can be attributed to their high selectivity and longer survival time in solution resulting in carbonate radicals reacting preferentially with electron-rich sites, such as aromatic rings.

The reaction of HPO_4_^2−^ with ^●^OH radicals is shown in Table 1, Equation (18). Although this reaction is rather slow, the positive effect on the NAB degradation can be explained by the difference in reactivity and selectivity of radicals towards NAB. Mártire et al. examined the reactivity and selectivity of HPO_4_^●−^, SO_4_^●−^, and ^●^OH radicals with various organic pollutants [30]. Their results indicate that, while phosphate radicals are less reactive than hydroxyl radicals, they exhibit significantly higher selectivity across all pollutants studied. Additionally, the overall reactivity trend observed is ^●^OH > SO_4_^●−^ > HPO_4_^●−^, while the selectivity trend is SO_4_^●−^ > HPO_4_^●−^ > ^●^OH.

Most studies on the degradation of pollutants have been carried out in pure aqueous solution, but the degradation efficiency can be influenced by different water matrices, including surface water, groundwater and wastewater, due to their complex composition. These matrices may contain various anions (such as Cl^−^, CO_3_^2−^, HCO_3_^−^, NO_3_^−^ and NO_2_^−^) as well as organic substances such as humic acid. Different water matrices can cause the differences in NAB degradation in comparison to NAB degradation in ultrapure water. An important focus of ongoing research is to understand the effects of radical scavengers, naturally occurring substances in the environment that interact with the pollutants being degraded. These radical scavengers, primarily anions and humic substances, compete with reactive species and can influence the degradation process.

**Figure 7:**
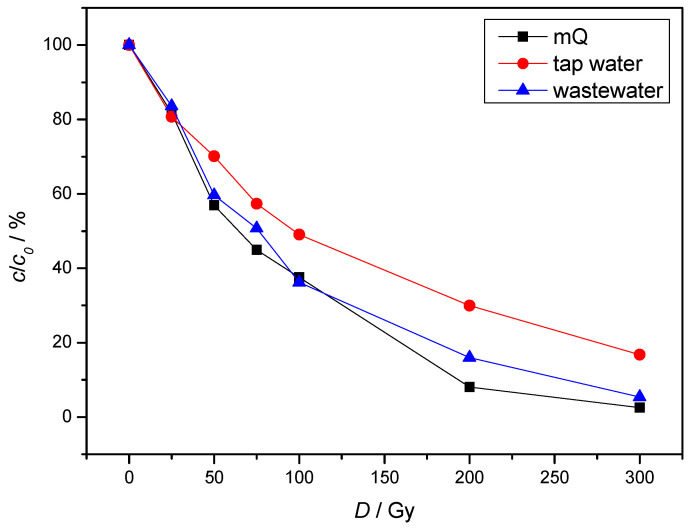
Radiolytic degradation of NAB dissolved in ultrapure water, tap water and synthetic wastewater under air equilibrium conditions. (*c*(NAB) = 2 × 10^−5^ mol/L, *P* = 0.4 Gy/s; synthetic wastewater composition: 6.96 mg (NH4)_2_SO_4_, 0.94 mg MgSO_4_, 7.05 mg K_2_HPO_4_, 7.01 mg humic acid, 81.37 mg NaHCO_3_ and 5.08 mg NAB in 1 L).

The gamma-radiolytic degradation of NAB was carried out in ultrapure water, wastewater and tap water. The degradation of NAB was slowest in tap water (Figure 7). With 200 Gy irradiation, 70.03, 84.03 and 91.94% NAB degradation was observed in tap water, wastewater and in ultrapure water respectively. The results obtained can be attributed to the fact that the inorganic ions present in the tap water react with the ^●^OH radicals, which reduces the possibility of their reaction with NAB. In contrast, the degradation efficiency in wastewater was increased. This is consistent with the previously published results of Zheng and co-authors [31]. This is due to the presence of humic acid, where triplet states of humic acid and various oxygen species can be formed after irradiation, increasing the concentration of radicals for reaction with NAB.

The results of these experiments, which were carried out under simulated conditions that can occur in the polluted environment, clearly show that the composition of the matrix has a significant influence on the degradation rate of micropollutants. The complete NAB degradation observed at an absorbed dose of about 300 Gy indicates that the radiolytic AOP process could be a competitive method for the degradation of pharmaceutical residues from wastewater compared to the methods routinely used today.

### 2.5. Identification of Degradation Products by LC-HRMS/MS

**Figure 8:**
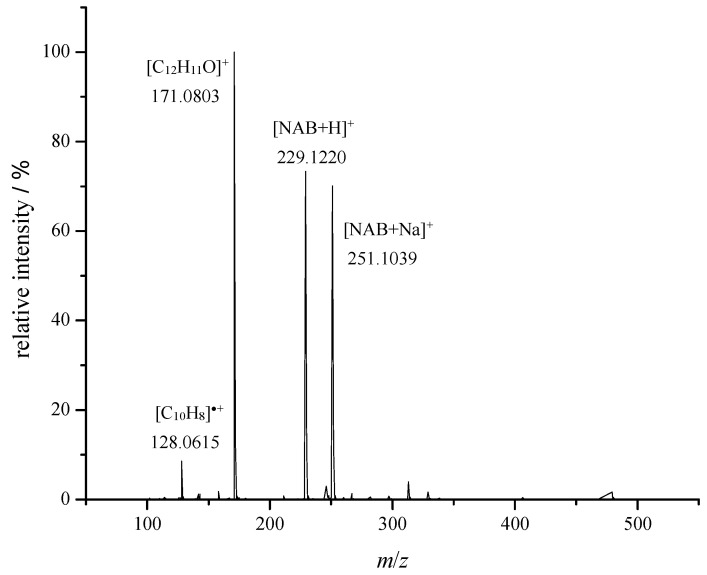
HRMS spectrum of NAB (*t*_R_ = 13.4 min).

Aqueous NAB solutions were irradiated with 150 Gy in the presence of air and N_2_O and analyzed by LC-HRMS. Eight degradation products were identified in the air saturated solution and fourteen in the N_2_O saturated solution. Structural characterization was performed based on the results of the tandem mass spectrometry analyses by collision-induced dissociation (CID). Detailed information on all identified degradation products is provided in Tables S1 and S2. The annotation of “a” and “b” refers to different experimental conditions, namely “a” stands for degradation in air saturated samples, while “b” for N_2_O saturated samples. The chromatographic peak of NAB was present in all chromatograms at the retention time of 13.4 min (Figure S3) with the characteristic signals at *m*/*z* 251 [M + Na]^+^, and 229 [M + H]^+^. Signals at *m*/*z* 171 and 128 were assigned to in-source fragments (Figure 8). MS/MS analysis was performed on the monoprotonated ion (Figure S4). Characteristic signals in the CID spectra of NAB were at *m*/*z* 128, 141, 156, and 171. Signals at *m*/*z* 128 and 156 with very low intensities were assigned to stable odd-electron radical ions C_10_H_8_^●+^ and C_12_H_12_^●+^ respectively [32,33]. A fragmentation pathway of NAB is proposed and can be found in the SI (Figure S5).

As indicated, ^●^OH radicals were the main reactive species responsible for NAB degradation. The absorption spectrum of aqueous NAB solution is characterized by the band at 231 nm resulting from the conjugated π-π* transition of the naphthalene ring. According to the UV-Vis spectrum shown in Figure 5, the intensity of the absorption band is significantly decreased with the increase of the absorbed dose, indicating disruption of aromatic conjugation in the naphthalene moiety of the NAB molecule. Similar results were reported by Chu et al. during the gamma radiolytic degradation of naphthalene in aqueous solution leading to the formation of naphthol intermediate and carboxylic acids such as formic and oxalic acid [34].

Analysis of two irradiated samples by HR MS showed different degradation products, with some similarities. The results are in agreement with those obtained by UV-Vis spectrophotometry, and published results on the degradation of naphthalene [34]. Degradation of NAB after gamma irradiation started mainly through hydroxylation of the naphthalene moiety with the formation of DP1a-3a and DP5a-6a in the case of air saturated solution, and DP1b, DP3b-5b, DP7b, DP10b-11b, and DP13b-14b in the case of N_2_O saturated solution as a result of NAB reaction with ^●^OH radicals. Presented HRMS data cannot unambiguously determine formation of favorable structures and their influence on subsequent ^●^OH oxidation steps of degradation. Extracted ion chromatograms (EIC) together with corresponding HRMS spectra of observed NAB degradation products are given in Figures S6–S27. The results of MS/MS analyses by CID on monoprotonated ions or in-source fragments (ISF) of NAB degradation products at 10 eV are shown in Figures S28–S51.

Several degradation products were observed both in the air saturated and N_2_O saturated samples. The monoprotonated ion of *m*/*z* 277 appeared both in the air saturated (DP2a) and N_2_O saturated samples (DP3b) at a retention time of 9.3 min. This degradation product corresponds to trihydroxylated NAB where one hydroxyl group is located on the naphthalene moiety. The structure of this degradation product was recently reported by us as a product of oxidative degradation by hydrogen peroxide at elevated temperature [35]. The monoprotonated ions of *m*/*z* 259 were also observed both in the air saturated and N_2_O saturated samples (DP5a, DP8a and DP7b, DP12b respectively).

The structure of DP3a (*m*/*z* 217) observed in the air saturated sample was also recently reported by us as a product of oxidative degradation [35].

The degradation product DP4b (*m*/*z* 261) observed in the N_2_O saturated sample shares a similar structure with one degradation product recently described by Tu et al. during the UV/monochloramine treatment of NAB aqueous solution [10]. They have suggested that both hydroxyl groups were at the naphthalene ring. However, based on the MS/MS results, we have suggested another structure of this degradation product with the same molecular formula. The difference in proposed structures could be the consequence of different experimental condition used (gamma irradiation vs UV/monochloramine treatment) for NAB degradation.

Six degradation products (DP1b, DP5b, DP10-11b, and DP13-14b) with the same *m*/*z* value of 245 assigned to the monoprotonated ion were observed at different retention times in the case of the N_2_O saturated sample. These degradation products, which are positional isomers, correspond to the monohydroxylated NAB molecule. They differ in the position of the hydroxyl group on the naphthalene moiety. Defining the exact position of the hydroxyl group is impossible based just on their MS/MS spectra alone as their monoprotonated ions share a similar fragmentation pathway. The monohydroxylated NAB degradation product was observed in the air saturated sample as well (DP7a). However, hydroxyl group was not located on the naphthalene moiety as in the aforementioned degradation products. Since the monoprotonated ion of this degradation product (*m*/*z* 245) was not present in its MS spectrum, structural characterization was performed based on MS/MS spectra of observed in-source fragments (*m*/*z* 227 and 185). The same was in case of DP9b which was observed in the N_2_O saturated sample. The degradation product DP8b observed in the N_2_O saturated sample was previously identified by us but in different degradation conditions. It was observed during the hydrolytic degradation of NAB acidic conditions [36]. Compound 4-(6-hydroxy-2-naphthtyl)-2-butanone was previously recognized as one of the pharmacologically inactive NAB metabolites [37,38].

### 2.6. Mineralization and Toxicity

**Figure 9:**
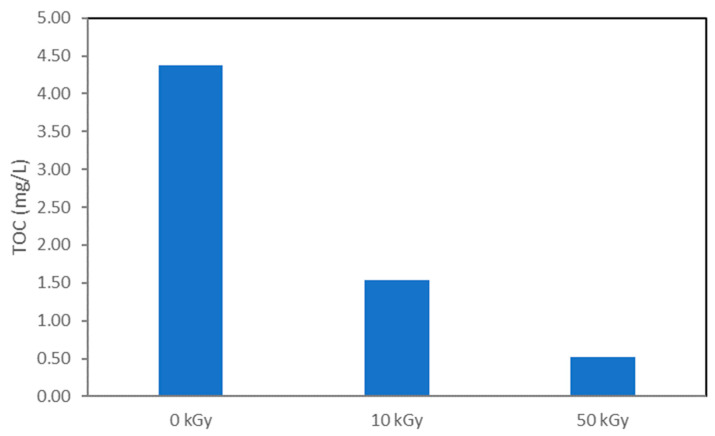
TOC (mg/L) as a function of the absorbed dose.

The value of total organic carbon (TOC) was determined from the experiments with higher absorbed doses [39], and the change in TOC during gamma irradiation is shown in Figure 9. The TOC values in the control solution were low due to the low NAB concentration used. The obtained results show that gamma-irradiation can lead both to degradation and partial mineralization of NAB (88% at 50 kGy) in aqueous solution. In practice, 50–60% TOC removal is generally sufficient to eliminate organics responsible for toxicity by forming short aliphatic chains such as carboxylic acids, which are known to be non-toxic and biodegradable. According to Zaouak and co-authors the energy cost to achieve 60% of mineralization of hydrochloroquinone with irradiation of 4 kGy was satisfactory compared to other AOPs such as electrochemical oxidation in combination with sonication and UV irradiation [40].

**Figure 10:**
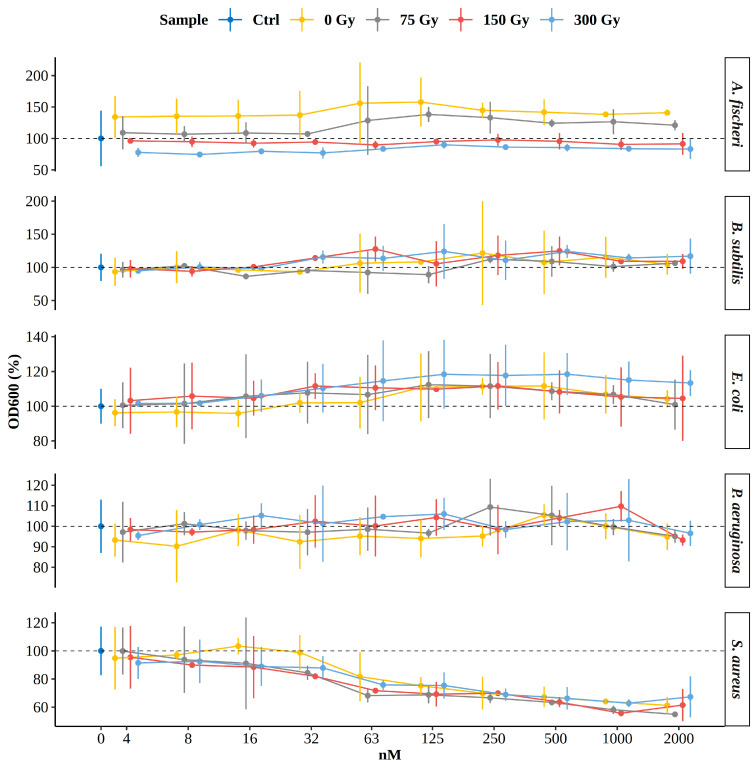
The influence of irradiated and non-irradiated NAB aqueous solution test samples on growth of five different bacteria by broth microdilution assay analyzed spectrophotometrically at wavelength of 600 nm (OD600).

Since the degradation products formed during AOPs can sometimes be more toxic than the parent molecule the antibacterial activity of NAB was further investigated. The influence of different absorbed doses on 10 NAB concentrations on the growth of five bacterial species is depicted in Figure 10. *A. fischeri* is frequently used in toxicity tests due to its sensitivity to a range of pollutants as an effective bioindicator for the toxicity of complex wastewater mixtures. In our case, the degradation products of NAB obtained under the experimental condition used, were found to be non-toxic. Interestingly, the inhibition of growth was only observed for *S. aureus* starting from ~63 nmol/L and was not affected by different doses of irradiation. Within the concentration range tested, there was no apparent concentration-response relationship of NAB (and the compounds induced by irradiation) as growth inhibition was fairly uniform for all four samples, averaging between ~50–75% between 125 and 2000 nmol/L. The results obtained are consistent with structurally related compounds that have a naphthyl moiety, such as naproxen. Naproxen and its derivatives have been reported to exhibit significant antibacterial properties, particularly against resistant bacterial strains, such as *S. aureus* [41,42]. Aromatic compounds can disrupt bacterial cell membranes, leading to membrane damage and loss of integrity, which is critical for bacterial survival. Although the primary function of NSAIDs is to relieve pain and inflammation, they may also have indirect antimicrobial effects. Selective antibacterial activity found in this study has also been observed with other non-steroidal anti-inflammatory drugs. For instance, ibuprofen and diclofenac, showed higher toxicity to *S. aureus* than to Gram-negative species *E. coli* and *P. aeruginosa* while aspirin exhibited a broad antibacterial range against both Gram-negative and positive pathogenic species and mefenamic acid showing no bacterial activity at all [43]. Similarly, variable effects on bacterial metabolism have been observed with environmental strains of soil microorganisms [36].

Growth is expressed relative to respective positive controls (inoculated media without test samples denoted as “Ctrl” and highlighted by dotted lines) for each species and shown only at 0 nmol/L concentration in blue. Four test samples are color coded and were tested at 10 different concentrations by two-fold dilution ranging from 2000 to 4 nmol/L. The dots represent the average values and the lines extend to +/−2 standard deviations of the respective replicates. The x-axis is log2 transformed and rounded for better readability.

### 2.7. Toxicity Estimates/Predictions of NAB and Degradation Products

Toxicity studies were extended with computational tools to predict in vivo toxicity in mammals. Prediction of oral rat toxicity of NAB, its transformation products and the toxicity of MNA was performed by the Toxicity Estimation Software Tool (TEST version 5.1) of the U.S. Environmental Protection Agency [44]. Oral rat toxicity is considered as the most important describing toxicity/non-toxicity potential of a compound. The reliability of oral rat toxicity prediction was improved in such a way that predictions were done/performed using two latest and commonly used programs for toxicity prediction Protox 3.0 [45] and ADMETLab 3.0 [46]. In addition, the highest and average predicted oral rat toxicity values for compounds with multiple isomers were calculated and presented to provide a more realistic representation of the toxicity of the products (Table 2). Similar to a recent work [10], activities/properties of compounds related to toxicity, such as bioaccumulation factor (BCF), developmental toxicity and mutagenicity, were also predicted by the TEST program.

For NAB (Table S3 and Table 2), program TEST provided an experimental oral rat LD50 value of 3877.26 mg/kg, which was higher than prediction by the TEST (2311.3 mg/kg) and higher than the prediction of all its degradation products. However, the prediction by ProTox 3.0 for NAB (3880 mg/kg) is very close to mentioned experimental value for NAB (see also in Table S3), indicating that NAB was probably included in the training set of ProTox 3.0 during the model development phase.

Among the degradation products from Table S1, the most toxic compound according to the prediction of oral rat toxicity by the TEST program is isomer 2 of DP1a (926 mg/kg), followed by isomer 6 of DP3a (1245.9 mg/kg) and DP4a (1252.6 mg/kg) (Table S3). All these compounds are predicted by the TEST program to be more toxic than NAB (2311.3 mg/kg) but less toxic than MNA (433.8 mg/kg).

The second method (ProTox 3.0) predicts isomer 5 of DP3a (348 mg/kg) as the most toxic product in terms of oral toxicity to rodents, followed by isomer 2 of DP2a (500 mg/kg) and isomer 1 of DP1a (530 mg/kg). Again, these predicted LD50 concentrations are lower (i.e., toxicities are higher) than those determined with ProTox 3.0 for NAB (3880 mg/kg), but lower than those predicted for MNA (248 mg/kg). According to the Pro-Tox 3.0 classification model defined on the basis of predicted rodent oral toxicity values, all degradation products from Table S1 belong to class 4 or 5 (the least toxic is class 6), NAB belongs to class 5, while MNA belongs to class 3. Similar results were obtained for the prediction of oral toxicity for rats using the AMETlab 3.0 method (Table S3), according to which all degradation products fall into the non-toxic or only slightly toxic category. The most toxic compound is predicted to be isomer 5 of DP3a (0.561, values on a scale of 0–1, where 0 indicates a non-toxic compound). All radiolytic degradation products from Table S1 are also predicted to be less toxic than MNA, and most of them are similar to the predicted toxicity of NAB.

The oral toxicity predictions of the TEST program for degradation products from Tables S2 and S4 show that the most toxic compound is DP6b (1181.12 mg/kg), followed by isomer 6 of DP7b (1329.84 mg/kg) and DP9b (1332.22 mg/kg) (Table S4). All products are predicted by the TEST program to be less toxic than the compounds in Tables S1 and S3, slightly more toxic than NAB (2311.3 mg/kg), but less toxic than MNA (433.8 mg/kg).

ProTox 3.0 predicts isomer 2 of DP3b (500 mg/kg) as the most toxic product in terms of oral toxicity to rodents, followed by DP2b (1190 mg/kg) and isomer 6 of DP4b (1500 mg/kg). These predicted toxicity values are lower (i.e., the toxicities are higher) than those determined with ProTox 3.0 for NAB (3880 mg/kg, class 5), but higher (i.e., the toxicities are lower) than those for MNA (248 mg/kg, class 3). According to the ProTox 3.0 classification model, which was defined on the basis of the predicted oral toxicity values for rodents, 23 degradation products from Table S4 belong to class 5 and only 7 products/isomers to toxicity class 4.

Predictions of oral toxicity to rats using the AMETlab 3.0 method for compounds from Table S4 show that 28 degradation products fall into the non-toxic category while only two into the slightly toxic category (DP2b and DP12b). The most toxic compound is DP2b (0.658, scale from 0–1, where 0 means a non-toxic compound). All degradation products from Tables S2 and S4 are also less toxic than MNA (0.692) and most of them are similar to the predicted oral rat toxicity of NAB (0.173).

The average oral LD40 toxicity predicted by the TEST program for the products in Table S4 (2210 mg/kg) is more than 20% higher than the average toxicity of the degradation products in Table S3 (1746.7 mg/kg). When predicting LD50 oral toxicity with the ProTox 3.0 method, the difference is even greater (2327 vs. 3126 mg/kg, difference ~30%). An analogous conclusion can be drawn from the predictions of ADMETlab 3.0. It therefore appears that the degradation products in Table S4 are (20–30)% less toxic than the corresponding products in Table S3. From the comparison of the minimum predicted LD50 values (Table 2), we see that for the first mechanism the minimum toxicity (763.1/666.0 mg/kg) is about 50% higher (lower LD50) than for the second one (1321.2/1044.6 mg/kg).

In general, all degradation products (including possible isomers) appear to be slightly more toxic than NAB, but also less toxic than MNA which is a normal metabolite of NAB in living organisms, or very commonly used drug ibuprofen, whose toxicity is predicted by the TEST and ProTox 3.0 methods to be 1520.72 mg/kg and 299 mg/kg, respectively. For example, experimentally measured oral toxicity of ibuprofen in rats is 636.09 mg/kg (US EPA, 2020, TEST program). The products obtained from this study were predicted by the TEST program to be slightly less toxic (926 mg/kg for DP1a and 1181.12 mg/kg for DP6b) than the products from Tu et al. [10] (839.54 mg/kg for MNA-P5 or 882.37 for MNA-P1), which are produced by UV/Monochloramine process.

The predictions of bioaccumulation factors (BCFs, unit L/kg) and developmental toxicity by the TEST program [44] are significantly less reliable than the predictions of oral toxicity. This is because the models were developed (trained and validated) for a much smaller number of compounds and consequently only cover a small chemical space (taking into account structural diversity) and can only be used for a very rough estimate. Therefore, all degradation products have predicted BCF values that lie between the values for NAB (58.22) and the values for MNA (8.58). It can be observed that all degradation products in Tables S3 and S4 have lower predicted BCF values than the parent compound NAB. For the compounds in Tables S3 and S4, the average BCF values are 11.9 and 11.4, respectively, which classifies them as compounds that accumulate poorly in living organisms. Non-bioaccumulative compounds have a BCF < 2000 and <1000, according to the EU REACH and US definition [47].

The developmental toxicity is a dimensionless value in the range 0–1 (1 stands for toxic, 0 for non-toxic). For all compounds in Tables S3 and S4, the TEST program predicts that they are developmentally toxic, and this also applies to the parent compound NAB and its metabolite MNA.

The DP1a isomers from Table S3 are predicted to be mutagenic by the TEST program (isomer 6 is predicted to be the most likely mutagen). The value separating the classes of mutagenic from non-mutagenic compounds is a predicted value of 0.5. In addition, the product DP6a (6 isomers) from Table S3 is also predicted to be potentially mutagenic. In Table S4, the first 6 isomers of DP1b are predicted to be potentially mutagenic, as are DP8b, DP9b, DP12b, isomer 5 of DP4b and two isomers of DP7b. For NAB, the predicted mutagenicity value is 0.47, i.e., very close to the threshold value of 0.5, and similar values are predicted for the amino acids arginine (0.46), glutamine (0.46) and vitamin A (0.42). The predicted value for MNA is 0.1 (non-mutagenic), similar to the highly toxic compound 2,3,7,8-tetrachlorodibenzo-P-dioxin (0.14). In summary, according to the predictions of the TEST program, the mutagenicity of the products was slightly increased compared to NAB, and similar results were obtained for products resulting from the UV/NH_2_Cl treatment of NAB [10].

## 3. Materials and Methods

**Figure 11:**
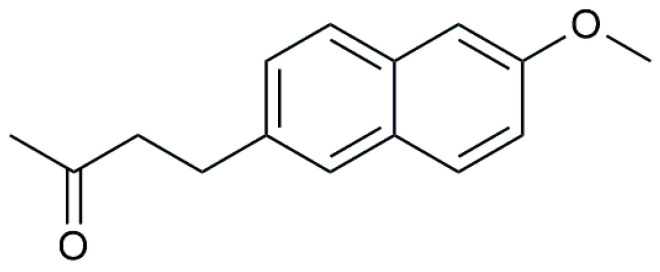
Chemical structure of nabumetone.

Nabumetone, the analytical standard, was provided by Cayman Chemical (Ann Arbor, MI, USA). The chemical structure is displayed in Figure 11. Chemicals for the HPLC-DAD analyses were as follows: acetic acid 100% p.a. from Honeywell Fluka (Seelze, Germany), ammonium acetate from Gram-mol (Zagreb, Croatia), acetonitrile (HPLC grade) from Sigma Aldrich (Saint-Quentin Fallavier, France). Potassium hydrogen phosphate and sodium hydroxide were purchased from Kemika (Zagreb, Croatia). Methanol and 2-propanol (HPLC grade) were purchased from Honeywell (Seelze, Germany). Sodium nitrate was obtained from Sigma Aldrich (Madrid, Spain) sodium hydrogen carbonate from Gram-Mol (Zagreb, Croatia), thiourea was purchased from Sigma and humic acid p.a. from Thermo Scientific (Erlenbachweg, Germany). Formic acid and acetonitrile (MS grade) for LC-MS analysis were purchased from Carlo Erba (Val-de-Reuil, France). Ultrapure water was obtained from the Mili-Q Advantage A10 purification system (Merck, Darmstadt, Germany). All chemicals were used as received without any further purification.

### 3.1. Irradiation

Aqueous solutions of NAB were prepared daily in concentrations 2 × 10^−5^ mol/L. The experiments were carried out in ultrapure water to allow the identification of products formed exclusively from the degradation of NAB and not from the reactions of NAB with other substances present in the environment. The pH of the unbuffered solutions was 6.4. The samples for the end-product experiments were irradiated in a panoramic type ^60^Co-γ irradiation chamber with different dose rates and with the following doses: 0, 25, 50, 75, 100, 200, and 300 Gy. Dose mapping of the irradiation facility has been performed experimentally (using ionizing chambers and ECB dosimetric system) and by simulation calculations [48]. The test solutions were in equilibrium with air or saturated with N_2_ or N_2_O in 15 mL ampoules (and thereafter airtight sealed) prior to the irradiation investigation. In the investigation of the hydrated electron reactions 2-propanol (2-PrOH) in concentration of 0.1 mol/L was used to scavenge the hydroxyl radicals. The temperature was kept constant at (20 ± 2) °C during the irradiation process. To investigate the effect of inorganic anions on the degradation of NAB, an aliquot of the stock solution was added prior to irradiation. The final concentration of NaHCO_3_ was 1.9 mmol/L, NaNO_3_ 1 mmol/L and K_2_HPO_4_ was 7 mg/L, respectively. All solutions were purged with N_2_. The matrix effect was studied using ultrapure water, tap water, and synthetic wastewater.

### 3.2. Preparation of Synthetic Wastewater

In order to investigate the influence of the aqueous matrix on the gamma-radiolytic degradation of NAB, synthetic wastewater was prepared in a volumetric flask according to the publication by Wojnárovits et al. [49]. The synthetic wastewater consisted of 6.96 mg (NH4)_2_SO_4_, 0.94 mg MgSO_4_, 7.05 mg K_2_HPO_4_, 7.01 mg humic acid, 81.37 mg NaHCO_3_ and 5.08 mg NAB in 1 L. The wastewater prepared in this way was sonicated in an ultrasonic bath for several hours. The measured pH of the prepared wastewater was 8.06. An aliquot of 5 mL was aerated with N_2_ for 15 min and exposed to gamma radiation at various doses (0.4 Gy/s).

### 3.3. HPLC-DAD Measurements and UV–Vis Spectrophotometry

Aqueous solutions of NAB were analyzed by HPLC-DAD KNAUER K-501 (Berlin, Germany). The separation was achieved using Nucleosil C18 column (5 μm, 4.0 × 250 mm; Macherey-Nagel, Düren, Germany). The column thermostat was maintained at 25 °C and the injection volume was 100 μL. The mobile phase consisted of acetonitrile (A) and ammonium acetate (10 mmol/L) (B). The ratio of eluent A and eluent B was changed from initial 80/20 to 20/80 over 15 min. The flow rate was 1.4 mL/min and the monitored wavelength was 231 nm.

The absorption spectra of NAB within irradiation time were recorded in the wavelength range of 200–400 nm using UV–Vis spectrophotometer, Varian Cary 4000 (Mulgrave, Victoria, Australia).

### 3.4. LC-HRMS/MS Measurements

High-resolution mass spectrometry analysis of degradation products was performed on Agilent 6550 Series Accurate-Mass-Quadrupole Time-of-Flight mass spectrometer coupled with Agilent 1290 Infinity II UHPLC (Santa Clara, CA, USA). The chromatographic separation was performed on Zorbax Eclipse Plus C18 (1.8 µm, 2.1 × 50 mm; Agilent, Santa Clara, CA, USA) column with the temperature set at 40 °C. The mobile phase consisted of 0.1% formic acid in water (A) and 0.1% formic acid in methanol (B) with the gradient elution profile as follows: 0 min 95% A; 15 min 5% A. The flow rate was set at 0.2 mL/min, and the injection volume was 2 µL. HRMS analyses were performed by electrospray ionization in positive mode with optimized parameters as follows: capillary voltage 2500 V; fragmentor voltage 100 V; sheath gas temperature 250 °C; sheath gas flow rate 11 L/min, drying gas temperature 200 °C; drying gas flow rate 17 L/min; nebulizer pressure 25 psi. Nitrogen was used as drying and sheath gas. Collision-induced dissociation was performed using collision energies (CE): 10, 20, 30, and 40 eV.

### 3.5. Toxicity Test

Untreated and samples treated with 75, 150, and 300 Gy were tested for antimicrobial activity against Gram-positive *Staphylococcus aureus* ATCC 6538 and *Bacillus subtilis* ATCC6633 and Gram-negative *Escherichia coli* NCTC 12241, *Pseudomonas aeruginosa* NCTC 12903, and *Aliivibrio fischeri* NRRL B-11177 bacteria using a broth microdilution method according to the CLSI guidelines [50] with minor modifications. Bacterial strains were recovered from −80 °C using tryptone soya agar (Biolab, Budapest, Hungary). Fresh overnight growth was used to prepare inocula to 0.5 McFarland by turbidity adjustment in 5 mL of sterile phosphate-buffered solution using a turbidimeter (bioMérieux, Marcy-l′Étoile, France) and thereafter diluted with cation-adjusted Mueller-Hinton broth (Merck, Darmstadt, Germany) to reach the final concentration of ≈5 × 10^5^ CFU/mL per well. Assays using *A. fischerii* were performed using Marine broth (HiMedia, Hessen, Germany). All assays employed positive (inoculated media without the tested sample) and negative (sterile media) control. All cultivations of bacteria and assays were performed aerobically at 35 °C except for *A. fischerii* at 28 °C. The concentration range tested was from 2000 nmol/L to 4 nmol/L by two-fold dilutions. The results were interpreted both visually and spectrophotometrically using a microplate reader (Tecan, Grödig, Austria). Plates were first shaken for 10 s in orbital mode with an amplitude of 3 mm and then absorbance was scanned at a wavelength of 600 nm (OD600) with 25 flashes after 100 ms of settle time with a microtiter lid in place. Minimal inhibitory concentration (MIC) is the lowest concentration that completely inhibits the growth of bacteria. Since no complete inhibition of bacterial growth was observed, the growth in each well was expressed as a percentage relative to the growth in respective positive control wells.

### 3.6. Toxicity Assessment Using Available QSAR Models

Acute toxicity (oral rat LD50, mg/kg), bioaccumulation factor (BCF), developmental toxicity (DevTox), and mutagenicity were predicted by TEST (Toxicity Estimation Software Tool, version 5.1) program [44] for NAB, its degradation products and MNA. The TEST program contains Quantitative Structure–Activity Relationship (QSAR) models developed for these four toxicity-related activities validated and used by many researchers and practitioners (e.g., Tu et al. [10]). In the TEST program, predictions are made using consensus models (ensemble) based on data sets of 7422 compounds for oral toxicity in rats, 676 for bioconcentration factor (BCF), 285 for developmental toxicity (DevTox) and 5735 for mutagenicity. These extensive data sets lend particular robustness to the models for oral toxicity in rats and for mutagenicity [44].

For substances whose molecular structures could not be accurately determined by mass spectrometry in a way that a single isomer is identified, all possible structures (Tables S1 and S2) and corresponding toxicity data were predicted and listed in Supplementary Tables (Tables S3 and S4). The minimum values are also marked in bold for each compound. The toxic doses are given as LD50 values in mg/kg body weight. The LD50 is the median lethal dose, i.e., the dose at which 50% of the test subjects die after exposure to a substance.

Prediction of oral toxicity was also made using the ProTox 3.0 method [45] for oral toxicity in rodents and the ADMETLab 3.0 method [46] for oral toxicity in rats. Both methods are very robust and were developed using a large number of chemicals (38,515 and 7327, respectively). More details on these methods (TEST, ProTox 3.0 and ADMETlab) are given in Note 2 in Supplementary Information (below Table S2).

## 4. Conclusions

The work presented here shows that ionizing radiation can be considered a suitable technique for the removal of NAB in water. At initial concentrations between 2 × 10^−5^ mol/L and 8 × 10^−6^ mol/L of NAB, degradation was 100% efficient at 300 Gy. The degradation reaction of NAB followed pseudo-first order kinetics. The aqueous matrix influenced the degradation rate of NAB, with NO_3_^−^ inhibiting the degradation of NAB and HPO_4_^2−^ and HCO_3_^−^ enhancing the degradation of NAB. The experiments with radical scavengers showed that ^●^OH radicals were responsible for the radiolytic degradation of NAB. Eight degradation products elucidated by LC-ESI-HRMS/MS were identified in an aqueous NAB solution in equilibrium with air and fourteen were detected when the solution was saturated with N_2_O. Toxicity studies on four different bacteria showed no significant change compared to the non-irradiated NAB. On the other hand, in silico studies showed that acute toxicity and developmental toxicity of degradation products of NAB increased after radiolytic treatment but were still lower than the toxicity of main NAB metabolite MNA. In future work, further studies need to be carried out focusing on the effects of ionizing radiation on the degradation of MNA and on the biotoxicity of the radiolytic products formed during AOP.

## Figures and Tables

**Table 1:** Chemical reactions and rate constants for radical scavengers and inorganic anions with different reactive species [19,22,23,24,25].

Reaction	*k*/mol^−1^ L s^−1^	Equation
^●^OH + ^●^H → H_2_O	7 × 10^9^	(4)
^●^OH + ^●^OH → H_2_O_2_	5.5 × 10^9^	(5)
^●^OH + e^−^_aq_ + ^●^H → H_2_ + OH^–^	2.5 × 10^10^	(6)
^●^OH + e^−^_aq_ → OH^−^	3.0 × 10^10^	(7)
e_aq_^−^ + N_2_O + H_2_O → ^●^OH + N_2_ + OH^−^	9.1 × 10^9^	(8)
^●^OH + (CH_3_)_2_COH → H_2_O + ^●^CH_2_CCH_3_OH	1.9 × 10^9^	(9)
^●^H + (CH_3_)_2_COH → H_2_ + ^●^CH_2_CCH_3_OH	7.4 × 10^7^	(10)
e^−^_aq_ + H^+^ → ^●^H	2.3 × 10^10^	(11)
^●^H + ^−^OH → e^−^_aq_ + H_2_O	2.2 × 10^7^	(12)
NO_3_^−^ + e^−^_aq_ → NO_3_^2−^	9.7 × 10^9^	(13)
H^+^ + NO_3_^−^ → HNO_3_	(4.4–6.0) × 10^8^	(14)
^●^OH + HNO_3_ → H_2_O + ^●^NO_3_	(0.88–1.2) × 10^8^	(15)
^●^H + HNO_3_ → H_2_ + ^●^NO_3_	1 × 10^7^	(16)
^●^OH + HCO_3_^−^ → CO_3_^•−^ + H_2_O	1.0 × 10^7^	(17)
^●^OH + HPO_4_ ^2−^ → HPO_4_^● −^ + OH^−^	8 × 10^5^	(18)

**Table 2:** Average values of minimal oral toxicities (LD50) for degradation products predicted by TEST and Pro-Tox 3.0 methods.

		LD50 (Avg. 1) ^(a)^	LD50 (Avg. 2) ^(a)^		LD50 (Avg. 1) ^(a)^	LD50 (Avg. 2) ^(a)^
		Table S3 ^(b)^	Table S3 ^(b)^		Table S4 ^(c)^	Table S4 ^(c)^
1	DP1a	763.1	726.6	DP1b	2769.8	2537.6
2	DP2a	1341.2	1044.6	DP2b	1321.2	1314.7
3	DP3a	801.0	666.0	DP3b	1341.2	1044.6
4	DP4a	2566.3	2204.6	DP4b	1549.0	1548.2
5	DP5a	2604.9	2271.5	DP5b ^(d)^	2769.8	2537.6
6	DP6a	1291.5	1291.2	DP6b	2530.6	2140.7
7	DP7a	3159.1	3075.8	DP7b	2604.9	2271.5
8	DP8a	3216.8	3147.7	DP8b	1506.6	1504.2
9				DP9b	2606.1	2273.5
10				DP10b ^(d)^	2769.8	2537.6
11				DP11b ^(d)^	2769.8	2537.6
12				DP12b	3216.8	3147.7
13				DP13b ^(d)^	2769.8	2537.6
14				DP14b ^(d)^	2769.8	2537.6
	NAB				3095.6	2994.6
	MNA				340.9	328.0
	HCN				21.1	13.6
	Minimal value ^(e)^	763.1	666.0		1321.2	1044.6

^(a)^ Average of LD50 oral toxicity predicted by TEST and Pro-Tox 3.0 methods obtained by (1) simple averaging of predicted concentrations named average 1 (avg. 1), and (2) by averaging predicted concentrations on the log10 scale named average 2 (avg. 2). For each Degradation Product (DP1a/DP1b, …, DP13b) having more than one isomer, minimal LD50 value was taken. ^(b)^ LD50 values related to compounds from Tables S1 and S3, i.e., to main degradation products for NAB in the air saturated solution. ^(c)^ LD50 values related to compounds from Tables S2 and S4, i.e., to main degradation products for NAB in N_2_O saturated solution. ^(d)^ Resulted in identical degradation products (isomers) as DP1b. ^(e)^ Minimum values of degradation products of rows 1–8 and 1–13 for the first and second mechanism, respectively.

## Data Availability

The original contributions presented in the study are included in the article/Supplementary Materials. Further inquiries can be directed to the corresponding author.

## Supplementary Materials

The following supporting information can be downloaded at: https://www.mdpi.com/article/10.3390/molecules30010064/s1, The SI material published online alongside the article includes the UV-Vis spectra of NAB (Figures S1 and S2), proposed structures of main degradation products analyzed by LC-HRMS in the presence of air and N_2_O (Tables S1 and S2); chromatograms and MS/MS spectra od NAB and its degradation products (Figures S3–S51); toxicity prediction of main degradation products (Tables S3 and S4).
