# Supplementary material for: Radiolytic Elimination of Nabumetone from Aqueous Solution: Degradation Efficiency, and Degradants’ Toxicity
Source: Molecules. 2024 Dec 27;30(1):64. doi: 10.3390/molecules30010064 (PMC11722061; doi:10.3390/molecules30010064)
Supplement: Supplementary file 1 [file molecules-30-00064-s001.zip › Supporting Information_revised.docx]

**Radiolytic elimination of nabumetone from aqueous solution: Degradation efficiency, and degradants' toxicity**

Ivana Tartaro Bujak, David Klarić, Bono Lučić, Krunoslav Bojanić, Maro Bujak, Nives Galić

**Supporting Information**

Contents

[1. UV-Vis spectroscopy 2](#_Toc185838807)

[2. Mass spectrometry 4](#_Toc185838808)

[3. Toxicity estimates/predictions of NAB and degradation products 29](#_Toc185838809)

# UV-Vis spectroscopy

**Figure S1**. UV-Vis spectra of NAB in N_2_ saturated aqueous solutions after irradiation.

*c*(NAB) = 2 × 10^−5^ mol/L. *P* = 0.4 Gy/s

**Figure S2**. UV-Vis spectra of NAB in aqueous solutions with 2-propanol after irradiation.

*c*(NAB) = 2 × 10^−5^ mol/L. *P* = 0.4 Gy/s

# 2. Mass spectrometry

**Table S1**. Determination of main degradation products for NAB in air saturated solution

|  | *t*_R_ / min | *m/z* values of characteristic MS-ESI (+) signals | mass error / ppm | molecular weight | molecular formula | proposed structural formula |
| --- | --- | --- | --- | --- | --- | --- |
| DP1a | 7.492 | 243.0627 – [M+Na]^+^  221.0807 – [M+H]^+^  203.0707 – ISF* | –0.33  –0.61 | 220.2240 | C_12_H_12_O_4_ |  |
| DP2a | 9.274 | 575.1874 – [2M+Na]^+^  299.0892 – [M+Na]^+^  277.1075 – [M+H]^+^  203.0704 – ISF* | –2.38  0.69  1.62 | 276.2880 | C_15_H_16_O_5_ |  |
| DP3a | 9.511 | 239.0673 – [M+Na]^+^  217.0860 – [M+H]^+^ | –2.36  0.37 | 216.2360 | C_13_H_12_O_3_ |  |
| DP4a | 9.781 | 267.0624 – [M+Na]^+^  245.0806 – [M+H]^+^  187.0752 – ISF* | –1.42  –0.96 | 244.2460 | C_14_H_12_O_4_ |  |
| DP5a | 10.288 | 539.1688 – [2M+Na]^+^  281.0788 – [M+Na]^+^  259.0975 – [M+H]^+^ | 2.15  1.32  3.92 | 258.2730 | C_15_H_14_O_4_ |  |
| DP6a | 11.036 | 269.0783 – [M+Na]^+^  247.0965 – [M+H]^+^ | –0.48  0.06 | 246.2620 | C_14_H_14_O_4_ |  |
| DP7a | 11.265 | 511.2087 – [2M+Na]^+^  267.0993 – [M+Na]^+^  227.1066 – ISF* | –0.80  0.50 | 244.2900 | C_15_H_16_O_3_ |  |
| DP8a | 11.892 | 539.1659 – [2M+Na]^+^  281.0782 – [M+Na]^+^  259.0964 – [M+H]^+^ | 2.15  –0.82  3.92 | 258.2730 | C_15_H_14_O_4_ |  |
| NAB | 13.428 | 251.1055 – [M+Na]^+^  229.1230 – [M+H]^+^  171.0811 – ISF*  128.0622 – ISF* | 4.97  3.03 | 228.2910 | C_15_H_16_O_2_ |  |

*ISF denotes in-source fragment

**Table S2**. Determination of main degradation products for NAB in N_2_O saturated solution

|  | *t*_R_ / min | *m/z* values of characteristic MS-ESI (+) signals | mass error / ppm | molecular weight | molecular formula | proposed structural formula |
| --- | --- | --- | --- | --- | --- | --- |
| DP1b | 8.561 | 245.1171 – [M+H]^+^  187.0754 – ISF* | 0.50 | 244.2900 | C_15_H_16_O_3_ |  |
| DP2b | 9.027 | 251.0680 – [M+Na]^+^  229.0858 – [M+H]^+^ | 0.54  –0.53 | 228.2470 | C_14_H_12_O_3_ |  |
| DP3b | 9.297 | 299.0890 – [M+Na]^+^  277.1073 – [M+H]^+^  203.0703 – ISF* | 0.02  0.90 | 276.2880 | C_15_H_16_O_5_ |  |
| DP4b | 9.492 | 283.0942 – [M+Na]^+^  261.1126 – [M+H]^+^  203.0705 – ISF* | 0.42  1.78 | 260.2890 | C_15_H_16_O_4_ |  |
| DP5b | 9.725 | 245.1173 – [M+H]^+^  187.0755 – ISF* | 0.32 | 244.2900 | C_15_H_16_O_3_ |  |
| DP6b | 9.858 | 253.0835 – [M+Na]^+^  231.1017 – [M+H]^+^  173.0600 – ISF* | –0.06  0.56 | 230.2630 | C_14_H_14_O_3_ |  |
| DP7b | 10.302 | 539.1675 – [2M+Na]^+^  281.0789 – [M+Na]^+^  259.0974 – [M+H]^+^ | –0.26  –0.82  3.53 | 258.2730 | C_15_H_14_O_4_ |  |
| DP8b | 10.897 | 237.0892 – [M+Na]^+^  215.1071– [M+H]^+^  157.0659 – ISF* | 2.53  2.06 | 214.2640 | C_14_H_14_O_2_ |  |
| DP9b | 11.075 | 515.2396 – [2M+Na]^+^  269.1158 – [M+Na]^+^  229.1228 – ISF*  171.0814 – ISF* | –1.57  3.66 | 246.3060 | C_15_H_18_O_3_ |  |
| DP10b | 11.345 | 511.2091 – [2M+Na]^+^  267.1011 – [M+Na]^+^  245.1178 – [M+H]^+^  187.0758 – ISF* | –0.02  7.24  2.36 | 244.2900 | C_15_H_16_O_3_ |  |
| DP11b | 11.649 | 267.0993 – [M+Na]^+^  245.1175 – [M+H]^+^  187.0754 – ISF* | 0.50  1.14 | 244.2900 | C_15_H_16_O_3_ |  |
| DP12b | 11.910 | 281.0789 – [M+Na]^+^  259.0970 – [M+H]^+^ | 1.67  1.99 | 258.2730 | C_15_H_14_O_4_ |  |
| DP13b | 11.981 | 267.0991 – [M+Na]^+^  245.1177 – [M+H]^+^  187.0752 – ISF* | –0.25  1.95 | 244.2900 | C_15_H_16_O_3_ |  |
| DP14b | 12.189 | 511.2092 – [2M+Na]^+^  267.1000 – [M+Na]^+^  245.1187 – [M+H]^+^  187.0763 – ISF* | 0.18  3.12  6.03 | 244.2900 | C_15_H_16_O_3_ |  |
| NAB | 13.427 | 251.1055 – [M+Na]^+^  229.1230 – [M+H]^+^  171.0811 – ISF*  128.0622 – ISF* | 4.97  3.03 | 228.2910 | C_15_H_16_O_2_ |  |

*ISF denotes in-source fragment


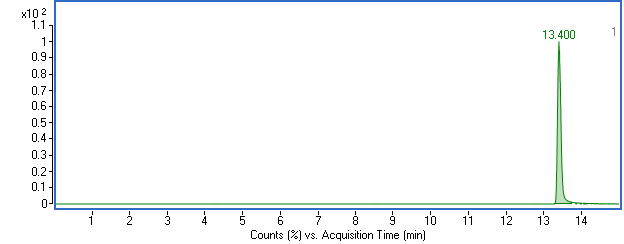


**Figure S3**. Extracted ion chromatogram of NAB.


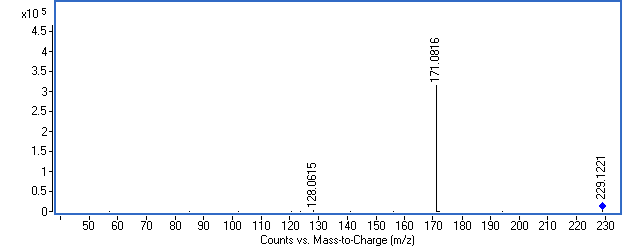


**Figure S4**. MS/MS spectrum of [NAB+H]^+^ (*m*/*z* 229.1220).

**Figure S5**. Fragmentation pathway of [NAB+H]^+^ (*m*/*z* 229.1220).


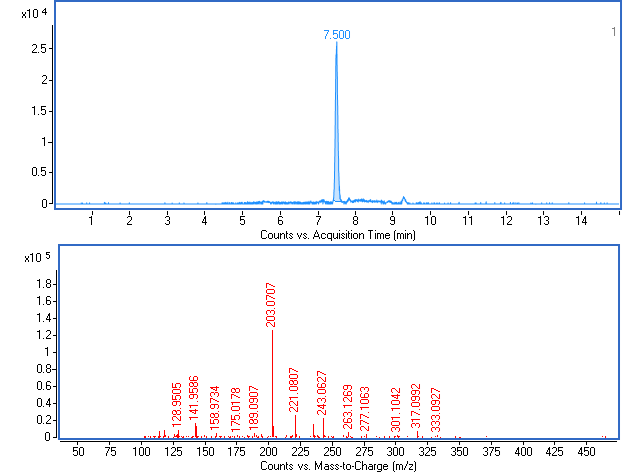


**Figure S6**. Extracted ion chromatogram (up) and MS spectrum (down) of DP1a.


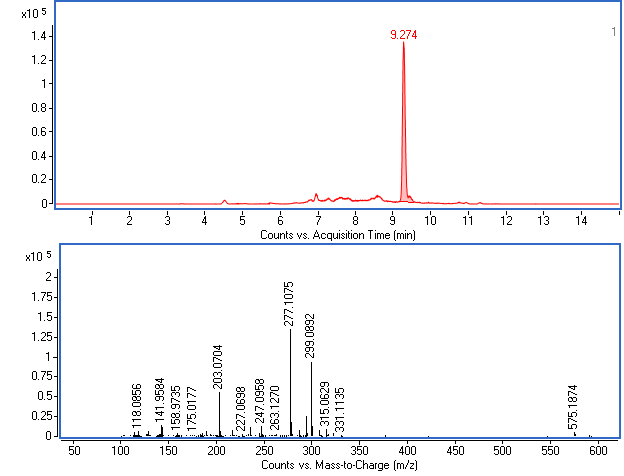


**Figure S7**. Extracted ion chromatogram (up) and MS spectrum (down) of DP2a.


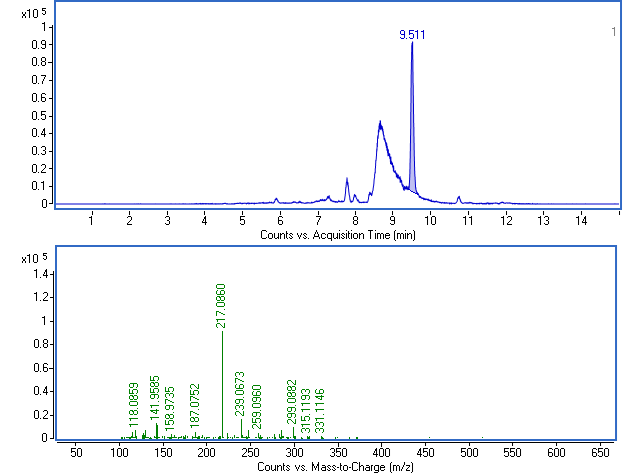


**Figure S8**. Extracted ion chromatogram (up) and MS spectrum (down) of DP3a.


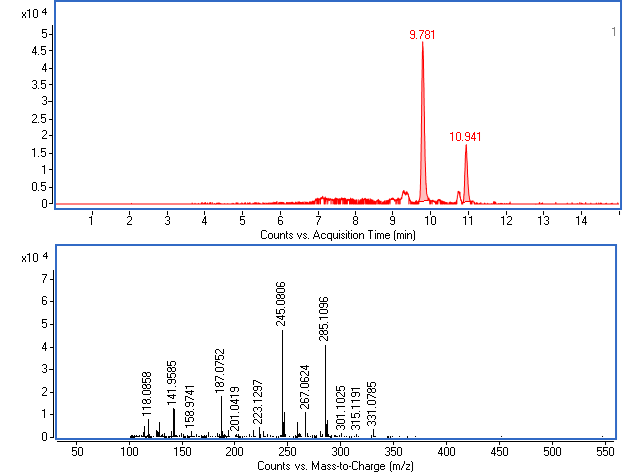


**Figure S9**. Extracted ion chromatogram (up) and MS spectrum (down) of DP4a.


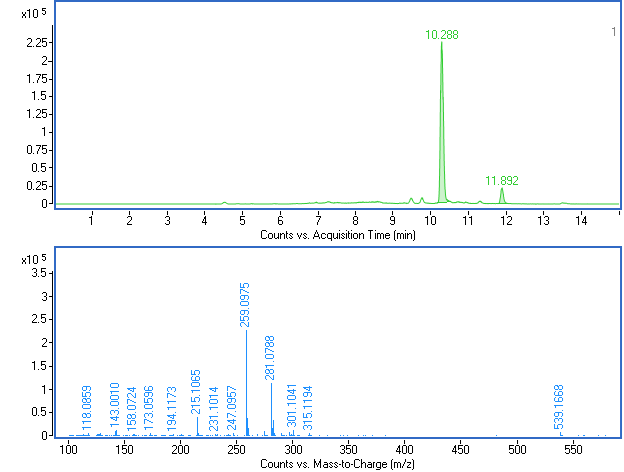


**Figure S10**. Extracted ion chromatogram (up) and MS spectrum (down) of DP5a.


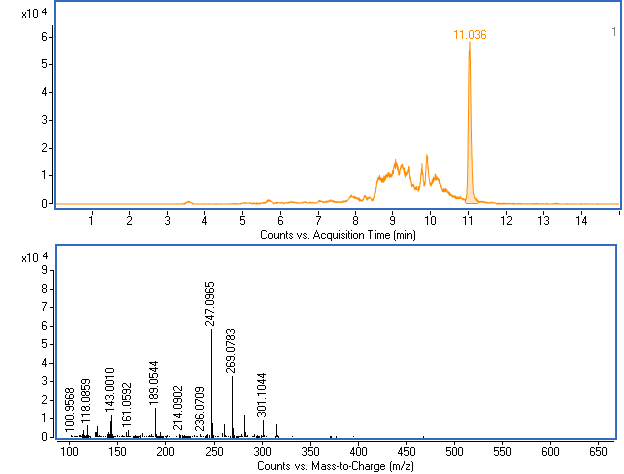


**Figure S11**. Extracted ion chromatogram (up) and MS spectrum (down) of DP6a.


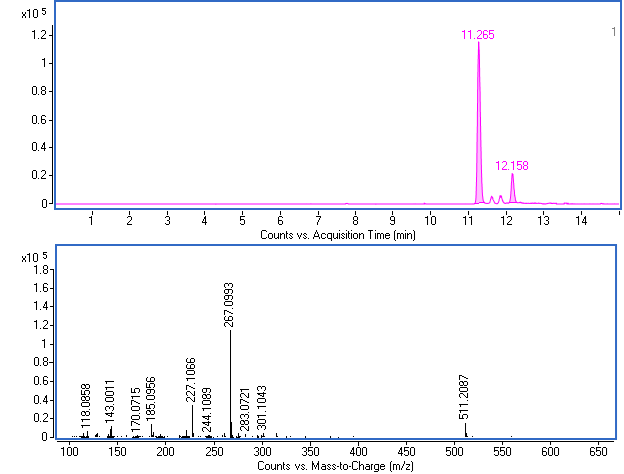


**Figure S12**. Extracted ion chromatogram (up) and MS spectrum (down) of DP7a.


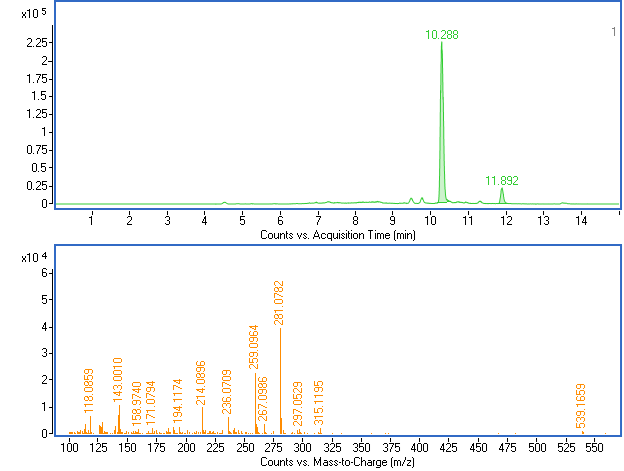


**Figure S13**. Extracted ion chromatogram (up) and MS spectrum (down) of DP8a.


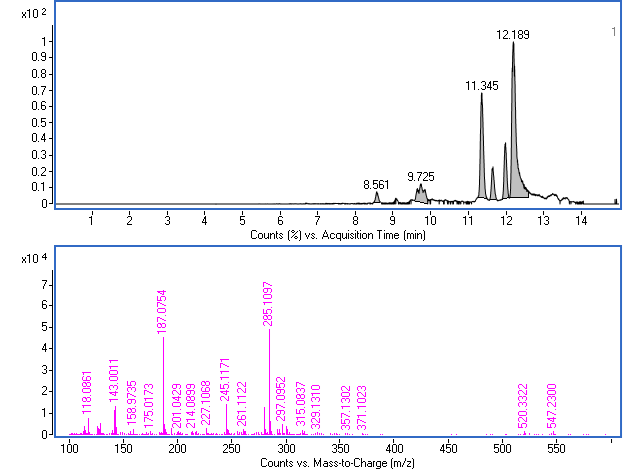


**Figure S14**. Extracted ion chromatogram (up) and MS spectrum (down) of DP1b.


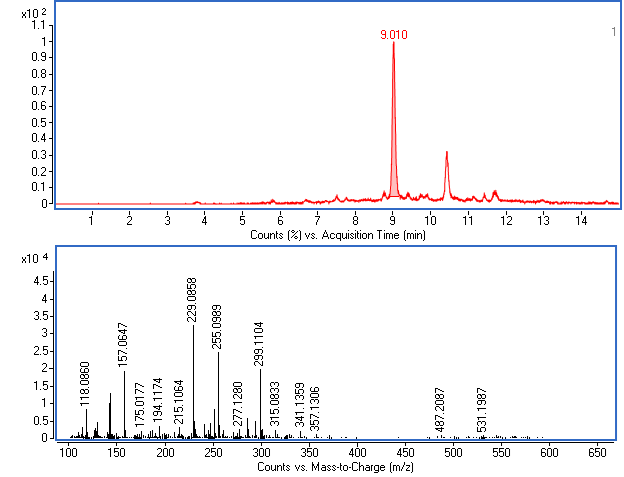


**Figure S15**. Extracted ion chromatogram (up) and MS spectrum (down) of DP2b.


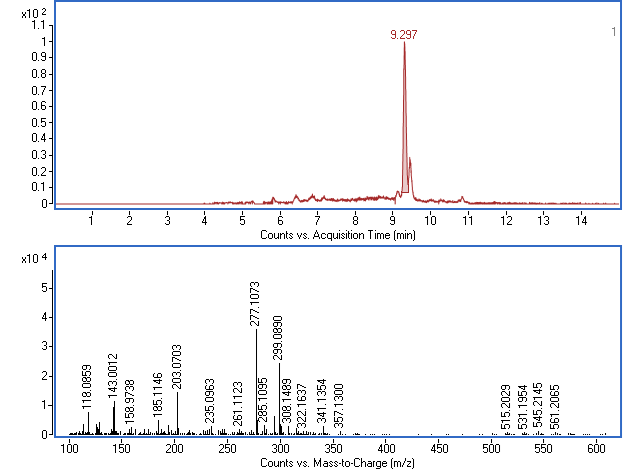


**Figure S16**. Extracted ion chromatogram (up) and MS spectrum (down) of DP3b.


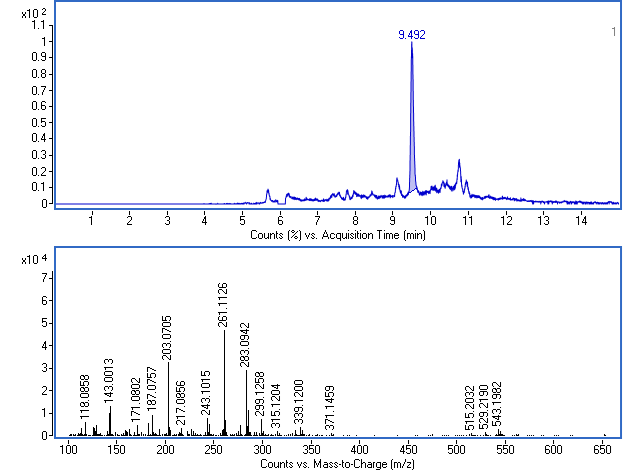


**Figure S17**. Extracted ion chromatogram (up) and MS spectrum (down) of DP4b.


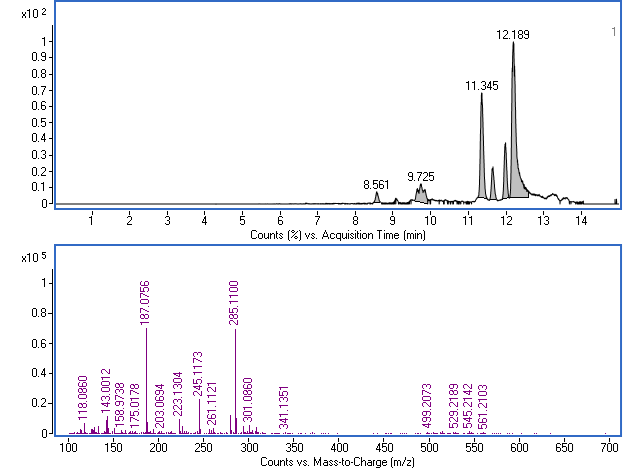


**Figure S18**. Extracted ion chromatogram (up) and MS spectrum (down) of DP5b.


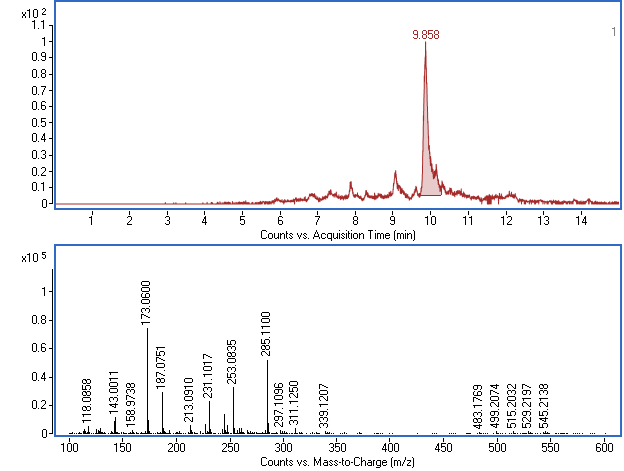


**Figure S19**. Extracted ion chromatogram (up) and MS spectrum (down) of DP6b.


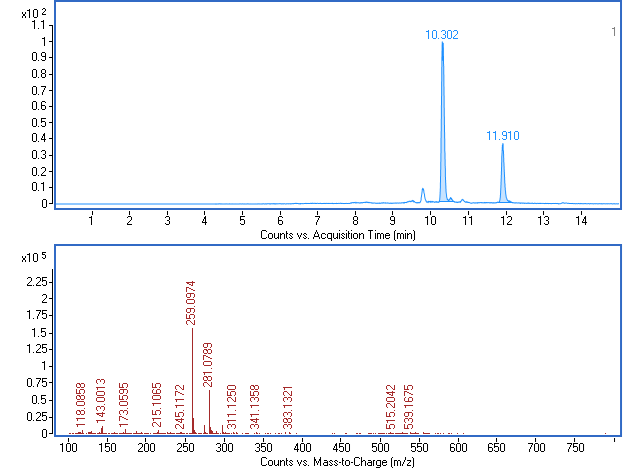


**Figure S20**. Extracted ion chromatogram (up) and MS spectrum (down) of DP7b.


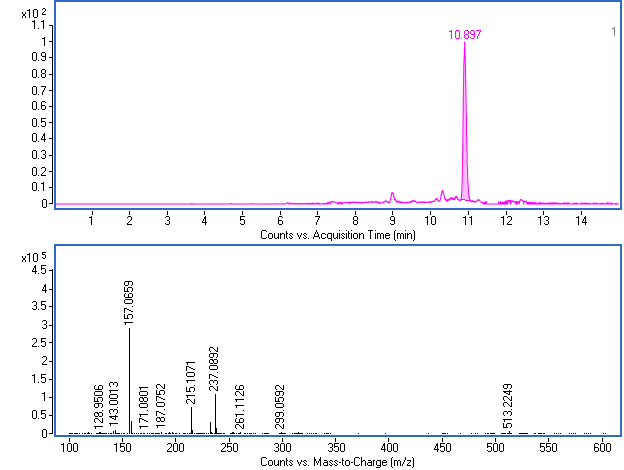


**Figure S21**. Extracted ion chromatogram (up) and MS spectrum (down) of DP8b.


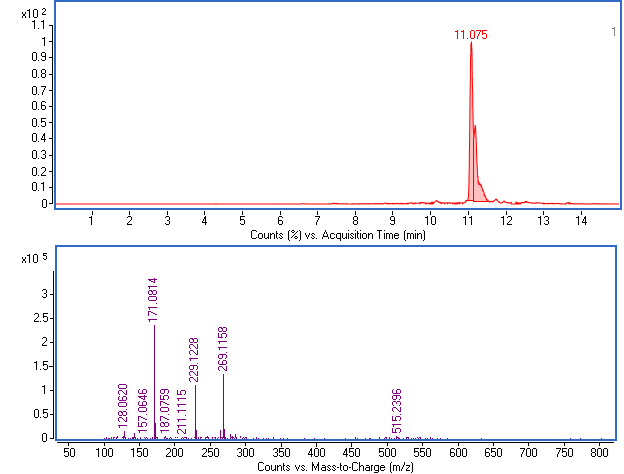


**Figure S22**. Extracted ion chromatogram (up) and MS spectrum (down) of DP9b.


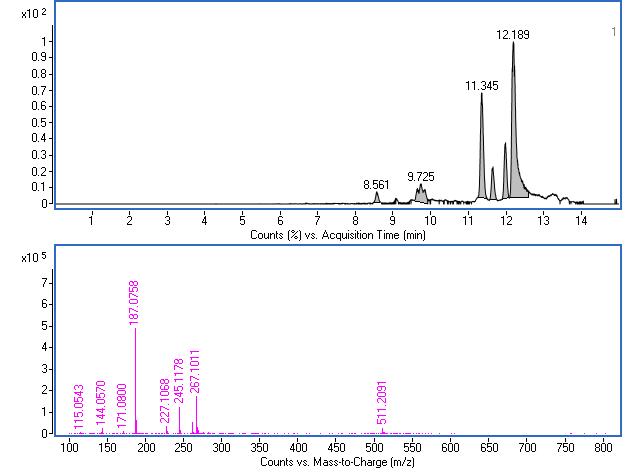


**Figure S23**. Extracted ion chromatogram (up) and MS spectrum (down) of DP10b.


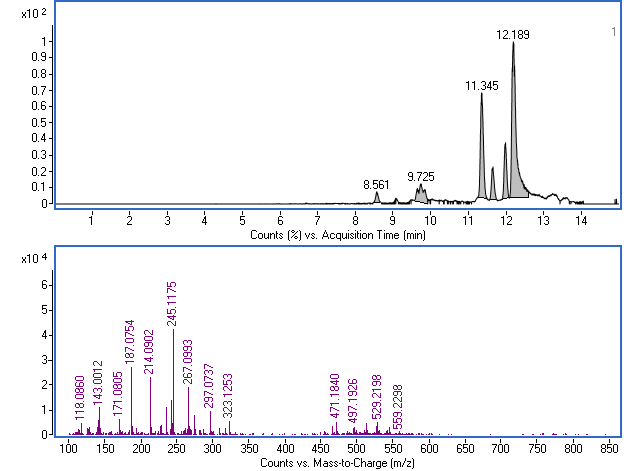


**Figure S24**. Extracted ion chromatogram (up) and MS spectrum (down) of DP11b.


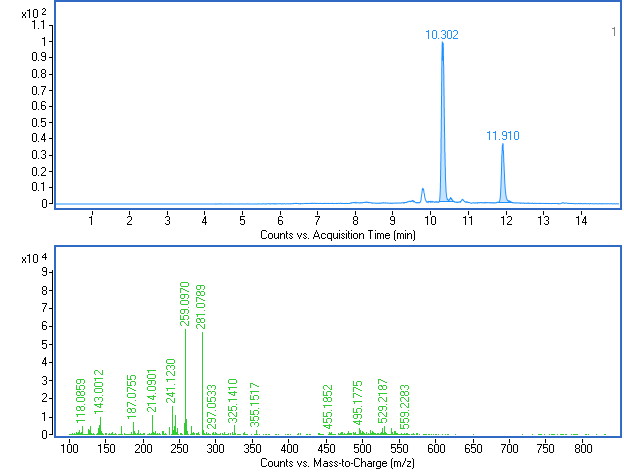


**Figure S25**. Extracted ion chromatogram (up) and MS spectrum (down) of DP12b.


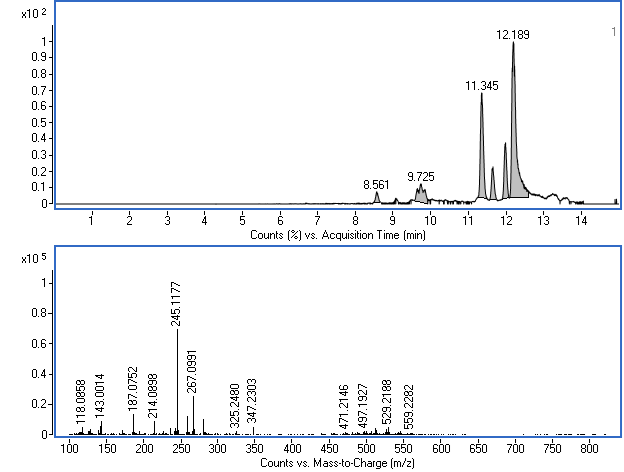


**Figure S26**. Extracted ion chromatogram (up) and MS spectrum (down) of DP13b.


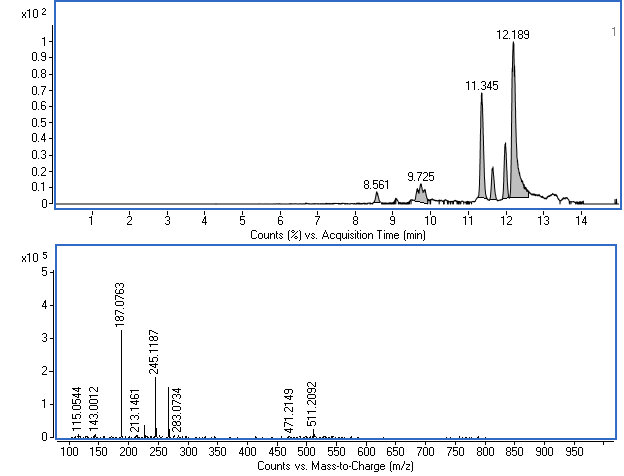


**Figure S27**. Extracted ion chromatogram (up) and MS spectrum (down) of DP14b.


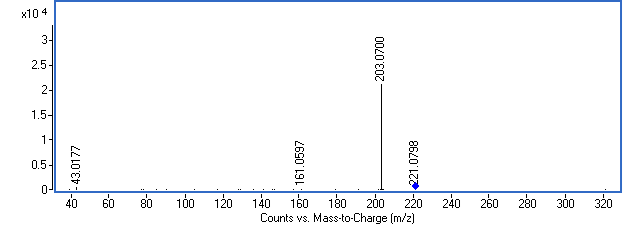


**Figure S28**. MS/MS spectrum of [NAB+DP1a]^+^ (*m*/*z* 221.0807).


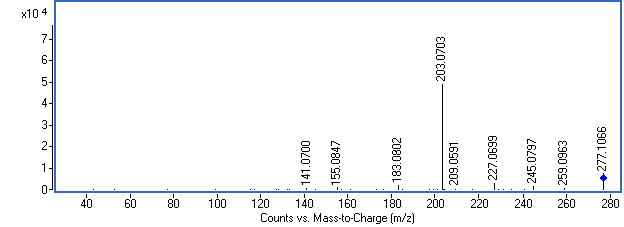


**Figure S29**. MS/MS spectrum of [NAB+DP2a]^+^ (*m*/*z* 277.1075).


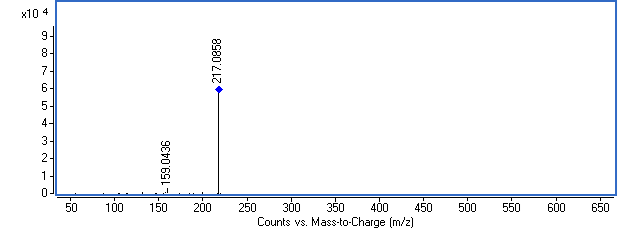


**Figure S30**. MS/MS spectrum of [NAB+DP3a]^+^ (*m*/*z* 217.0806).


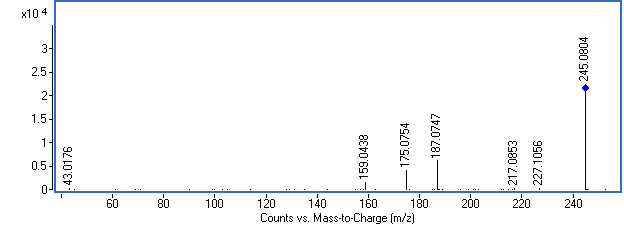


**Figure S31**. MS/MS spectrum of [NAB+DP4a]^+^ (*m*/*z* 245.0806).


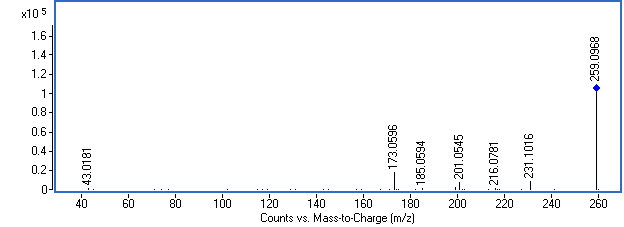


**Figure S32**. MS/MS spectrum of [NAB+DP5a]^+^ (*m*/*z* 259.0975).


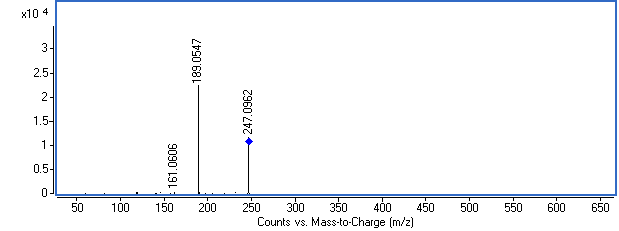


**Figure S33**. MS/MS spectrum of [NAB+DP6a]^+^ (*m*/*z* 247.0965).


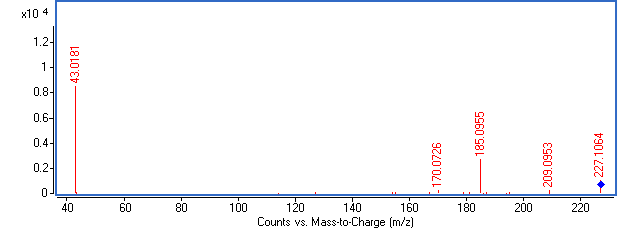


**Figure S34**. MS/MS spectrum of DP7a ISF (*m*/*z* 227.1066).


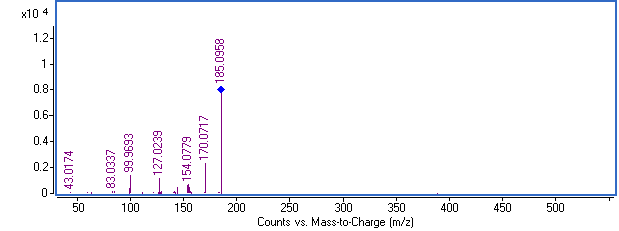


**Figure S35**. MS/MS spectrum of DP7a ISF (*m*/*z* 185.0956).


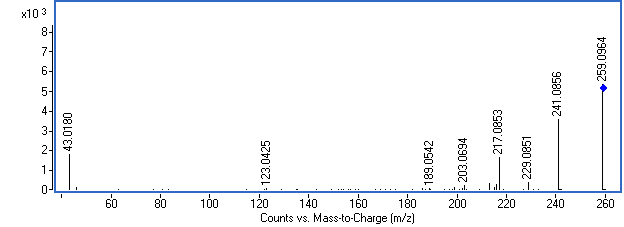


**Figure S36**. MS/MS spectrum of [NAB+DP8a]^+^ (*m*/*z* 259.0964).


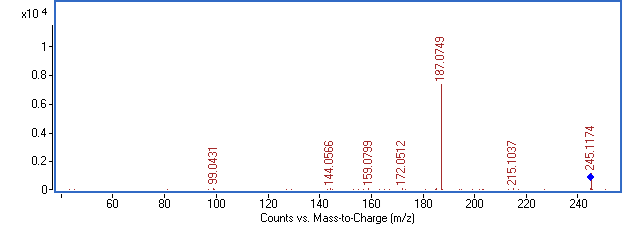


**Figure S37**. MS/MS spectrum of [NAB+DP1b]^+^ (*m*/*z* 245.1171).


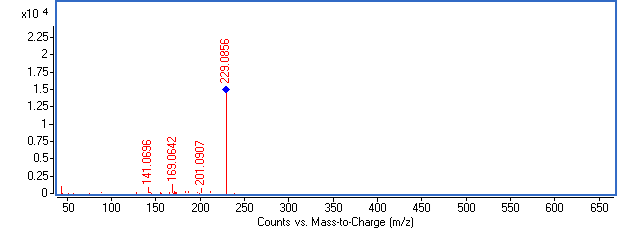


**Figure S38**. MS/MS spectrum of [NAB+DP2b]^+^ (*m*/*z* 229.0858).


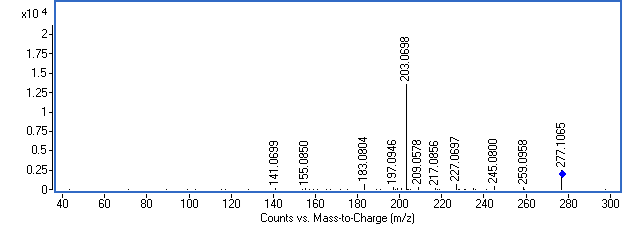


**Figure S39**. MS/MS spectrum of [NAB+DP3b]^+^ (*m*/*z* 277.1073).


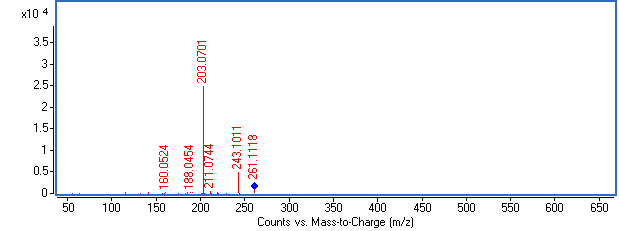


**Figure S40**. MS/MS spectrum of [NAB+DP4b]^+^ (*m*/*z* 261.1126).


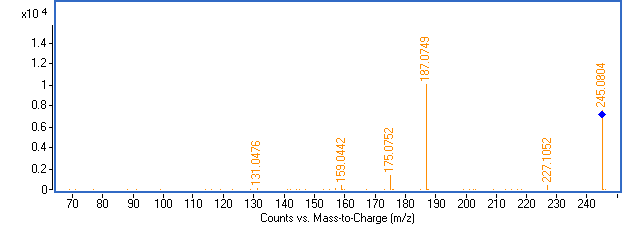


**Figure S41**. MS/MS spectrum of [NAB+DP5b]^+^ (*m*/*z* 245.1173).


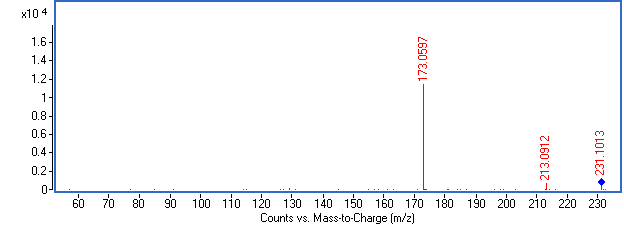


**Figure S42**. MS/MS spectrum of [NAB+DP6b]^+^ (*m*/*z* 231.1017).


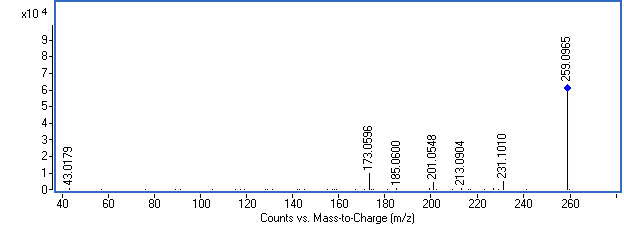


**Figure S43**. MS/MS spectrum of [NAB+DP7b]^+^ (*m*/*z* 259.0974).


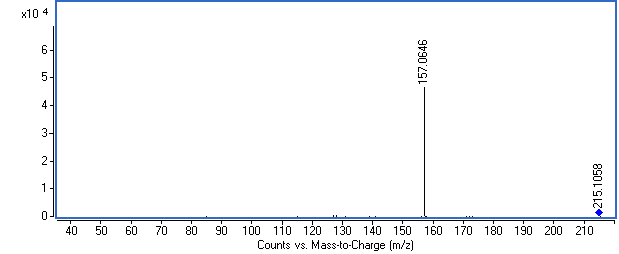


**Figure S44**. MS/MS spectrum of [NAB+DP8b]^+^ (*m*/*z* 215.1071).


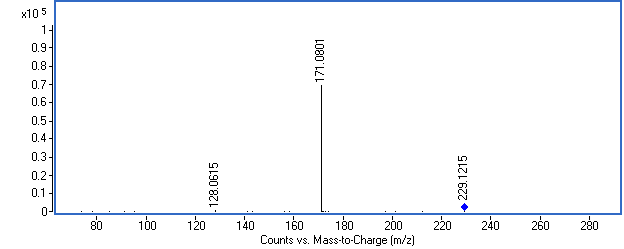


**Figure S45**. MS/MS spectrum of DP9b ISF (*m*/*z* 229.1228).


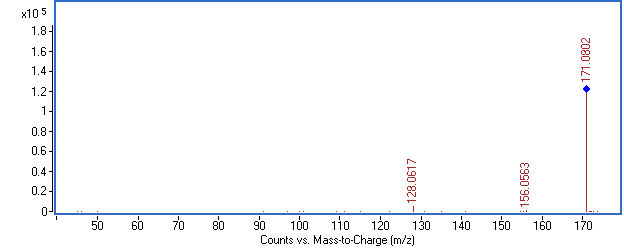


**Figure S46**. MS/MS spectrum of DP9b ISF (*m*/*z* 171.0814).


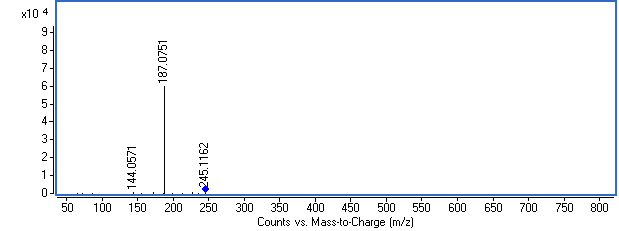


**Figure S47**. MS/MS spectrum of [NAB+DP10b]^+^ (*m*/*z* 245.1178).


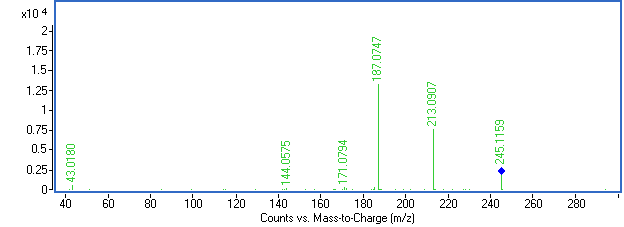


**Figure S48**. MS/MS spectrum of [NAB+DP11b]^+^ (*m*/*z* 245.1175).


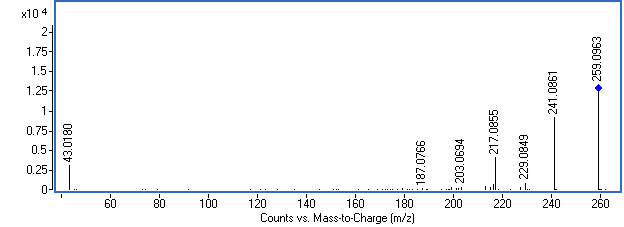


**Figure S49**. MS/MS spectrum of [NAB+DP12b]^+^ (*m*/*z* 259.0970).


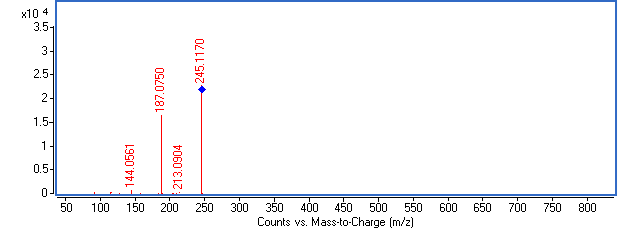


**Figure S50**. MS/MS spectrum of [NAB+DP13b]^+^ (*m*/*z* 245.1177).


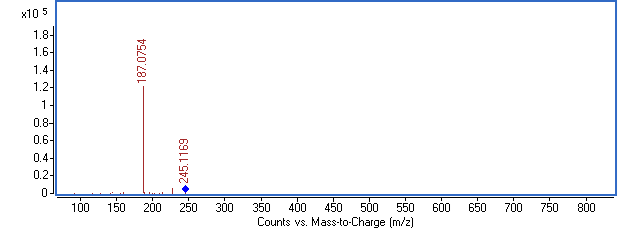


**Figure S51**. MS/MS spectrum of [NAB+DP14b]^+^ (*m*/*z* 245.1187).

# 3. Toxicity estimates/predictions of NAB and degradation products

**Note 1.**

**Table S3**. Toxicity prediction of main degradation products for NAB in air saturated solution

**Table S4**. Toxicity prediction of main degradation products for NAB in N_2_O saturated solution

Tables S3 and S4 are organized and given as MS Excel documents.

**Note 2. More information about the methods used for toxicity prediction (TEST, ProTox 3.0, ADMETlab)**

The individual toxicity models in the TEST program are based on calculated molecular descriptors and similarities between molecules. Further details on the individual models (hierarchical clustering, single model, group contribution model and the k-nearest neighbors (kNN method) that form the consensus model can be found in the supporting materials for the TEST program (Tables S3 and S4) in reference [20] given in the main text of the manuscript.

There is compatibility and overlap between the sets of compounds whose oral toxicity has been measured in rodents and rats, so that these predictions can be considered comparable in a crude assessment of toxicity that can be given with the models available today. The ProTox 3.0 method is based on the kNN method and fingerprints (FP24) and was validated by cross-validation, while the ADMETlab method uses the Directed Message Passing Neural Network (DMPNN) method.
